# Winner's Curse Correction and Variable Thresholding Improve Performance of Polygenic Risk Modeling Based on Genome-Wide Association Study Summary-Level Data

**DOI:** 10.1371/journal.pgen.1006493

**Published:** 2016-12-30

**Authors:** Jianxin Shi, Ju-Hyun Park, Jubao Duan, Sonja T. Berndt, Winton Moy, Kai Yu, Lei Song, William Wheeler, Xing Hua, Debra Silverman, Montserrat Garcia-Closas, Chao Agnes Hsiung, Jonine D. Figueroa, Victoria K. Cortessis, Núria Malats, Margaret R. Karagas, Paolo Vineis, I-Shou Chang, Dongxin Lin, Baosen Zhou, Adeline Seow, Keitaro Matsuo, Yun-Chul Hong, Neil E. Caporaso, Brian Wolpin, Eric Jacobs, Gloria M. Petersen, Alison P. Klein, Donghui Li, Harvey Risch, Alan R. Sanders, Li Hsu, Robert E. Schoen, Hermann Brenner, MGS (Molecular Genetics of Schizophrenia) GWAS Consortium, GECCO (The Genetics and Epidemiology of Colorectal Cancer Consortium), The GAME-ON/TRICL (Transdisciplinary Research in Cancer of the Lung) GWAS Consortium, PRACTICAL (PRostate cancer AssoCiation group To Investigate Cancer Associated aLterations) Consortium, PanScan Consortium, The GAME-ON/ELLIPSE Consortium, Rachael Stolzenberg-Solomon, Pablo Gejman, Qing Lan, Nathaniel Rothman, Laufey T. Amundadottir, Maria Teresa Landi, Douglas F. Levinson, Stephen J. Chanock, Nilanjan Chatterjee

**Affiliations:** 1 Division of Cancer Epidemiology and Genetics, National Cancer Institute, Bethesda, Maryland, United States of America; 2 Department of Statistics, Dongguk University, Seoul, Korea; 3 Center for Psychiatric Genetics, Department of Psychiatry and Behavioral Sciences, North Shore University Health System Research Institute, University of Chicago Pritzker School of Medicine, Evanston, Illinois, United States of America; 4 Dept. of Statistics, Northern Illinois University, DeKalb, Illinois, United States of America; 5 Information Management Services, Inc., Rockville, Maryland, United States of America; 6 Institute of Population Health Sciences, National Health Research Institutes, Miaoli, Taiwan; 7 Usher Institute of Population Health Sciences and Informatics, The University of Edinburgh, Medical School, Edinburgh, United Kingdom; 8 Department of Preventive Medicine and Department of Obstetrics and Gynecology, USC Keck School of Medicine, University of Southern California, Los Angeles, California, United States of America; 9 Norris Comprehensive Cancer Center, USC Keck School of Medicine, University of Southern California, Los Angeles, California, United States of America; 10 Genetic and Molecular Epidemiology Group, Spanish National Cancer Research Centre (CNIO), Madrid, Spain; 11 Geisel School of Medicine, Dartmouth College, Hanover, New Hampshire, United States of America; 12 Human Genetics Foundation, Turin, Italy; 13 MRC-PHE Centre for Environment and Health, School of Public Health, Imperial College London, London, United Kingdom; 14 National Institute of Cancer Research, National Health Research Institutes, Zhunan, Taiwan; 15 Department of Etiology & Carcinogenesis, Cancer Institute and Hospital, Chinese Academy of Medical Sciences and Peking Union Medical College, Beijing, China; 16 State Key Laboratory of Molecular Oncology, Cancer Institute and Hospital, Chinese Academy of Medical Sciences and Peking Union Medical College, Beijing, China; 17 Department of Epidemiology, School of Public Health, China Medical University, Shenyang, China; 18 Saw Swee Hock School of Public Health, National University of Singapore, Singapore; 19 Division of Molecular Medicine, Aichi Cancer Center Research Institute, Chikusa-ku, Nagoya, Japan; 20 Department of Preventive Medicine, Seoul National University College of Medicine, Seoul, Republic of Korea; 21 Department of Medical Oncology, Dana-Farber Cancer Institute, Boston, Massachusetts, United States of America; 22 Department of Medicine, Brigham and Women’s Hospital and Harvard Medical School, Boston, Massachusetts, United States of America; 23 Epidemiology Research Program, American Cancer Society, Atlanta, Georgia, United States of America; 24 Division of Epidemiology, Department of Health Sciences Research, Mayo Clinic, Rochester, Minnesota, United States of America; 25 Department of Oncology, the Johns Hopkins University School of Medicine, Baltimore, Maryland, United States of America; 26 Department of Epidemiology, the Bloomberg School of Public Health, Baltimore, Maryland, United States of America; 27 Department of Gastrointestinal Medical Oncology, University of Texas M.D. Anderson Cancer Center, Houston, Texas, United States of America; 28 Department of Chronic Disease Epidemiology, Yale School of Public Health, New Haven, Connecticut, United States of America; 29 Public Health Sciences Division, Fred Hutchinson Cancer Research Center, Seattle, Washington, United States of America; 30 Department of Medicine and Epidemiology, University of Pittsburgh Medical Center, Pittsburgh, Pennsylvania, United States of America; 31 Division of Clinical Epidemiology and Aging Research, German Cancer Research Center (DKFZ), Heidelberg, Germany; 32 Division of Preventive Oncology, German Cancer Research Center (DKFZ) and National Center for Tumor Diseases (NCT), Heidelberg, Germany; 33 German Cancer Consortium (DKTK), German Cancer Research Center (DKFZ), Heidelberg, Germany; 34 Department of Psychiatry and Behavioral Sciences, Stanford University, Stanford, California, United States of America; 35 Department of Biostatistics, Bloomberg School of Public Health, Johns Hopkins University, Baltimore, Maryland, United States of America; 36 Department of Oncology, School of Medicine, Johns Hopkins University, Baltimore, Maryland, United States of America; Institute for Molecular Medicine Finland (FIMM), FINLAND

## Abstract

Recent heritability analyses have indicated that genome-wide association studies (GWAS) have the potential to improve genetic risk prediction for complex diseases based on polygenic risk score (PRS), a simple modelling technique that can be implemented using summary-level data from the discovery samples. We herein propose modifications to improve the performance of PRS. We introduce threshold-dependent winner’s-curse adjustments for marginal association coefficients that are used to weight the single-nucleotide polymorphisms (SNPs) in PRS. Further, as a way to incorporate external functional/annotation knowledge that could identify subsets of SNPs highly enriched for associations, we propose variable thresholds for SNPs selection. We applied our methods to GWAS summary-level data of 14 complex diseases. Across all diseases, a simple winner’s curse correction uniformly led to enhancement of performance of the models, whereas incorporation of functional SNPs was beneficial only for selected diseases. Compared to the standard PRS algorithm, the proposed methods in combination led to notable gain in efficiency (25–50% increase in the prediction R^2^) for 5 of 14 diseases. As an example, for GWAS of type 2 diabetes, winner’s curse correction improved prediction R^2^ from 2.29% based on the standard PRS to 3.10% (*P* = 0.0017) and incorporating functional annotation data further improved R^2^ to 3.53% (*P* = 2×10^−5^). Our simulation studies illustrate why differential treatment of certain categories of functional SNPs, even when shown to be highly enriched for GWAS-heritability, does not lead to proportionate improvement in genetic risk-prediction because of non-uniform linkage disequilibrium structure.

## Author Summary

Large GWAS have identified tens or even hundreds of common SNPs significantly associated with individual complex diseases; however, these SNPs typically explain a small proportion of phenotypic variance. Recently, heritability analyses based on GWAS data suggest that common SNPs have the potential to explain substantially larger fraction of phenotypic variance and to improve the genetic risk prediction. Because of the polygenic nature, improving genetic risk prediction for complex diseases typically requires substantially increasing the sample size in the discovery set. Thus, it is crucial to develop more efficient algorithms using existing GWAS summary data. In this article, we extend the polygenic risk score (PRS) method by adjusting the marginal effect size of SNPs for winner’s curse and by incorporating external functional annotation data. Theoretical analysis and simulation studies show that the performance improvement depends on the genetic architecture of the trait, sample size of the discovery sample set and the degree of enrichment of association for SNPs annotated as “high-prior” and the linkage disequilibrium patterns of these SNPs. We applied our method to the summary data of 14 GWAS. Our method achieved 25–50% gain in efficiency (measured in the prediction R^2^) for 5 of 14 diseases compared to the standard PRS.

## Introduction

Large genome-wide association studies (GWAS) have accelerated the discovery of dozens or even hundreds of common single nucleotide polymorphisms (SNPs) associated with individual complex traits and diseases, such as height [1, 2], body mass index [3] and common cancers (e.g., breast [4] and prostate [5] cancers). Although individual SNPs typically have small effects, cumulative results have provided insight about underlying biologic pathways and for some common diseases like breast cancer have yielded levels of risk-stratification that could be useful as part of prevention efforts [6]. Analyses of GWAS heritability using algorithms such as GCTA [7, 8] have shown that common SNPs have the potential to explain substantially larger fraction of the variation of many traits.

The future yield of GWAS studies, for both discovery and prediction, depends heavily on the underlying effect-size distribution (ESD) of susceptibility SNPs [9, 10]. A number of alternative types of analyses of ESD now point towards a polygenic architecture for most complex traits, in which thousands or even tens of thousands of common SNPs, each with small estimated effect sizes together can explain a substantial fraction of heritability [11, 12]. Mathematical analyses of power indicates that because of the polygenic nature of complex traits, future studies will need large sample sizes, often by an order of magnitude higher than even some of the largest studies to date, for improving accuracy of genetic risk-prediction [10, 11]. Nevertheless, for current datasets, there remains an opportunity to develop more efficient algorithms for improving the models [13].

Available algorithms for polygenic risk score (PRS) prediction models have varying degrees of complexity. The simplest of these methods, widely implemented in large GWAS, selects SNPs based on a threshold for the significance of the marginal association test-statistics and then the cumulative weighting of these SNPs by their estimated marginal strength of association is applied [14]. The threshold for SNP selection can be optimized to improve the predictive performance in an independent validation dataset. For a number of traits with large GWAS sample sizes, it has been shown that an optimally selected threshold can improve risk prediction compared to that based on the genome-wide significance threshold used for discovery [15]. A number of newer methods involving the joint analysis of all SNPs using sophisticated mixed-effect modeling techniques have recently been developed and may lead to further increases in model performance [16–18].

In this report, we propose simple modifications to the widely used PRS modeling techniques using only GWAS summary-level data. Drawing from the lasso [19] algorithm, we propose a simple threshold dependent winner’s curse adjustment for marginal association coefficients that can be used to weight the SNPs in PRS. Second, to exploit external functional knowledge that might identify subsets of SNPs highly enriched for association signals, we consider using multiple thresholds for SNPs selection based on group membership and identify an optimal set of thresholds through an independent validation dataset. We demonstrated the value of our new method using summary-level results from large GWAS across a spectrum of traits, some with available independent validation datasets to assess the performance of these methods. Available resources, such as annotation databases, expression and methylation quantitative trait locus (QTL) analyses were employed to identify groups of SNPs that are likely to be enriched with the trait of interest. We evaluated the utility of this information for risk-prediction for respective outcomes. We also report on the performance of new algorithm using simulation studies that incorporate realistic genetic architecture, linkage disequilibrium pattern and enrichment factor for underlying functional SNPs.

## Results

### Overview of statistical approach

Let *Z*_*m*_, *P*_*m*_, ${\hat{\beta }}_{m}$, and ${\hat{\sigma }}_{m}$ (*m* = 1, …, *T*) denote the *Z*-statistics, the two-sided *P*-values, the estimated association coefficients and their standard deviations available as part of summary-level results for *T* SNPs from a GWAS. We assume that each genotypic value is normalized to have mean zero and unit variance and that ${\hat{\beta }}_{m}$ is rescaled to correspond to the normalized genotypic values. We assume that *M* SNPs are selected after LD-clumping, a SNP pruning procedure guided by the association *P*-values [20]. Let *g*_*im*_ be the genotype of SNP *m* for subject *i*. The simplest and most popular form of the PRS has the form

$$PRS_{i}(\alpha )=\sum_{m=1}^{M}{\hat{\beta }}_{m}I(P_{m}<\alpha )g_{im},$$ (1)

where the threshold *α* for the *P*-values can be chosen to optimize the predictive performance of PRS in an independent validation dataset. Here, *I* (⋅) is an indicator function. Because *PRS*_*i*_(*α*) uses a single threshold to select SNPs, we refer this as one-dimensional PRS or 1D PRS. In what follows, we extend *PRS*_*i*_(*α*) by incorporating annotation data and correcting for the upward bias in ${\hat{\beta }}_{m}$ caused by winner’s curse.

#### 2D PRS

Information from various functional studies, annotation databases and GWAS from various traits is increasingly available to allow identification of subset of SNPs that can be considered to have potential high-prior probability for association with a given trait. Various types of enrichment analyses, whether based on distribution of summary-level statistics [21] or on more advanced heritability-partitioning analyses [22, 23], have shown empirical evidence of strong enrichment of GWAS association signals for different categories of SNPs which represent only a relatively small fractions of all GWAS SNPs. However, very few systematic studies have examined whether and how such enrichment information can be utilized to improve models for genetic risk prediction. We consider a simple modification to PRS to explore this issue. We assume that the set of *M* SNPs can be partitioned into two mutually exclusive groups, *S*_*1*_ and *S*_*2*_, where *S*_*1*_ represents a relatively small subset representing “high-prior” SNPs (referred to as HP) and the second group *S*_*2*_ represents the remainder of the GWAS SNPs (referred to as “low-prior” SNPs or LP) that can be considered part of an “agnostic” search. We allow differential treatment of the SNPs in the PRS:

$$PRS_{i}(\alpha_{1},\alpha_{2})=\sum_{m\in S_{1}}^{}{\hat{\beta }}_{m}I(P_{m}<\alpha_{1})g_{im}+\sum_{m\in S_{2}}^{}{\hat{\beta }}_{m}I(P_{m}<\alpha_{2})g_{im}$$ (2)

and select the optimal (*α*_1_, *α*_2_) based on independent validation dataset(s). Intuitively, SNPs in the HP group are included at a less rigorous threshold than SNPs in the LP group to optimize the performance. We refer to the PRS in Eq (2) as two-dimensional PRS or 2D PRS.

**Fig 1:**
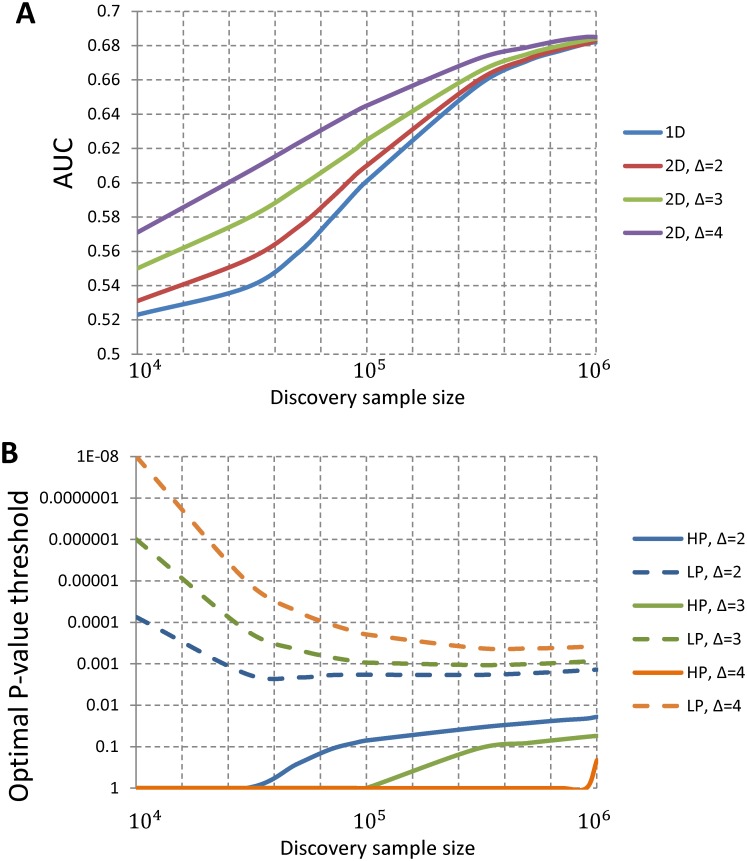
Theoretic investigation of prediction performance and optimal thresholds for SNP selection in 2D PRS. The theoretic calculation assumes *M* = 53,163 independent SNP, of which 5,000 are causal for a binary trait, similar to simulation studies. The high-prior (HP) SNP set has 5,000 SNPs and the low-prior (LP) SNP set has 48,163 SNPs. *Δ* is the enrichment fold of HP SNPs in the causal SNP set. (A) The prediction AUC for 1D PRS and 2D PRS. (B) The optimal P-value thresholds for including HP and LP SNPs in 2D PRS. For both plots, x-coordinate is the discovery sample size, assuming equal number of cases and controls.

When the genetic architecture parameters are known and SNPs are independent, we derived the theoretical predictive performance of 2D PRS and the corresponding optimal (*α*_1_, *α*_2_) following analytic techniques similar to those derived for 1D PRS [11] (Materials and Methods). Fig 1A shows the theoretically-derived area under the curve (AUC) for a binary trait based on 1D PRS and 2D PRS. For both PRS models, the AUC increases with the sample size of the discovery dataset. The 2D PRS can improve the 1D PRS in which the magnitude depends on the sample size in the discovery sample and also the enrichment fold change Δ of the HP SNPs. Here, Δ is defined as the ratio of the proportion of causal SNPs in HP to the overall proportion of causal SNPs. A larger value of Δ indicates a greater enrichment of causal SNPs in HP. Fig 1B shows the optimal *P*-value thresholds (*α*_1_, *α*_2_) for including SNPs that maximize the prediction of 2D PRS for a given sample size in the discovery sample. The optimal *P*-value threshold for including HP SNPs is more liberal than that for LP SNPs and the difference diminishes as the training sample size becomes very large.

#### PRS with winner’s curse correction

In PRS, only SNPs with *P*-values less than a specific threshold are included. This selection affects the probability density of ${\hat{\beta }}_{m}$ for selected SNPs and may cause upward bias in the estimate, an effect called winner’s curse. Methods have been proposed to reduce the selection bias in GWAS [24–26]; however, it is not clear whether winner’s curse corrections improve the performance of PRS. Let *β*_*m*_ denote the true effect size and assume that ${\hat{\beta }}_{m}\sim N(\beta_{m},{\hat{\sigma }}_{m}^{2})$. Following Zhong and Prentice [26], we consider a shrinkage estimator ${\hat{\beta }}_{m}^{mle}(\alpha )$ that maximizes a conditional likelihood

$$P({\hat{\beta }}_{m}|P_{m}<\alpha )=\frac{\phi (({\hat{\beta }}_{m}-\beta_{m})/{\hat{\sigma }}_{m})/{\hat{\sigma }}_{m}}{\Phi (\beta_{m}/{\hat{\sigma }}_{m}-\lambda /{\hat{\sigma }}_{m})+\Phi (-\beta_{m}/{\hat{\sigma }}_{m}-\lambda /{\hat{\sigma }}_{m})}I(|\beta_{m}|\geq \lambda (\alpha )),$$

where *ϕ*() is the density function of *N*(0,1), Φ() is the cumulative distribution function of *N*(0,1) and $\lambda (\alpha )=\Phi^{-1}(1-\alpha /2){\hat{\sigma }}_{m}$. The 1D PRS and 2D PRS after winner’s curse correction are calculated as

$$PRS_{i}^{mle}(\alpha )=\sum_{m=1}^{M}{\hat{\beta }}_{m}^{mle}(\alpha )I(P_{m}<\alpha )g_{im}$$ (3)

and

$$PRS_{i}^{mle}(\alpha_{1},\alpha_{2})=\sum_{m\in S_{1}}^{}{\hat{\beta }}_{m}^{mle}(\alpha_{1})I(P_{m}<\alpha_{1})g_{im}+\sum_{m\in S_{2}}^{}{\hat{\beta }}_{m}^{mle}(\alpha_{2})I(P_{m}<\alpha_{2})g_{im},$$ (4)

respectively. Because ${\hat{\beta }}_{m}^{mle}(\alpha )$ is a maximum likelihood estimator, we denote it as MLE winner’s curse correction. It is critical that for selection of the optimal threshold parameter(s), bias correction is performed simultaneously with SNP selection for different values of the threshold parameters. This approach, although conceptually straightforward, is computationally extensive for analyzing a large number of SNPs and a grid of *P*-value thresholds.

A computationally more attractive approach is to build a PRS using lasso [19] based on summary level data from a GWAS. Suppose that we have *M* independent SNPs and *N* training samples with phenotype *y*_*j*_. We assume that genotypic values *g*_*jm*_ are standardized to have mean zero and unit variance. We estimate parameters (*β*_0_, *β*_1_, …, *β*_*M*_) by minimizing a penalized loss function:

$$\frac{1}{2}{\sum_{j=1}^{N}\left(y_{j}-\beta_{0}-\sum_{m=1}^{M}\beta_{m}g_{jm}\right)}^{2}+\lambda \sum_{m=1}^{M}\left|\beta_{m}\right|,$$ (5)

where *λ* controls the sparseness of the prediction model. Let ${\hat{\beta }}_{m}=\sum_{j=1}^{N}\left(y_{j}-\bar{y}\right)g_{im}$ be the marginal estimate of *β*_*m*_. When SNPs are independent, the solution to Eq (5) was derived as [19]

$${\hat{\beta }}_{m}^{lasso}(\lambda )=sign({\hat{\beta }}_{m})||{\hat{\beta }}_{m}|-\lambda |I(|{\hat{\beta }}_{m}|>\lambda ).$$ (6)

The resulting linear prediction model, or equivalently the PRS, is given as

$$PRS_{i}^{lasso}(\lambda )=\sum_{m=1}^{M}{\hat{\beta }}_{m}^{lasso}(\lambda )g_{im}=\sum_{m=1}^{M}sign({\hat{\beta }}_{m})||{\hat{\beta }}_{m}|-\lambda |I(|{\hat{\beta }}_{m}|>\lambda )g_{im}.$$

Because event {*P*_*m*_ < *α*} is equivalent to event $\left\{|{\hat{\beta }}_{m}|>\lambda (\alpha )\right\}$ with $\lambda (\alpha )=\Phi^{-1}(1-\alpha /2)sd({\hat{\beta }}_{m})$, we can rewrite $PRS_{i}^{lasso}(\lambda )$ as

$$PRS_{i}^{lasso}(\alpha )=\sum_{m=1}^{M}sign({\hat{\beta }}_{m})||{\hat{\beta }}_{m}|-\lambda (\alpha )|I(P_{m}<\alpha )g_{im}.$$ (7)

Similarly, considering the lasso problem with two penalty terms by minimizing

$$\frac{1}{2}{\sum_{i=1}^{N}\left(y_{i}-\beta_{0}-\sum_{m=1}^{M}\beta_{m}g_{im}\right)}^{2}+\lambda_{1}\sum_{m\in S_{1}}|\beta_{m}|+\lambda_{2}\sum_{m\in S_{2}}\left|\beta_{m}\right|$$

leads to a 2D PRS

$$PRS_{i}^{lasso}(\alpha_{1},\alpha_{2})=\sum_{m\in S_{1}}^{}{\hat{\beta }}_{m}^{lasso}(\lambda (\alpha_{1}))I(P_{m}<\alpha_{1})g_{im}+\sum_{m\in S_{2}}^{}{\hat{\beta }}_{m}^{lasso}(\lambda (\alpha_{2}))I(P_{m}<\alpha_{2})g_{im}.$$ (8)

Note that the above derivation assumes independence between SNPs. In reality, nearby SNPs may still be in weak LD even after aggressive LD-clumping using *r*^2^ < 0.1. Thus, Eq (6) approximates the exact lasso solution that formally adjusts for correlation. The similarity between $PRS_{i}^{mle}(\alpha )$ in Eq (3) and $PRS_{i}^{lasso}(\alpha )$ in Eq (7) suggests that the lasso shrinkage estimator Eq (6) provides an alternative approach for reducing the bias caused by winner’s curse. This observation motivated us to use the shrinkage estimator in Eq (6) to build PRS for a binary trait, where ${\hat{\beta }}_{m}$ is marginally estimated. Because the models in Eqs (7) and (8) are approximations to the true lasso prediction model in presence of weak LD between SNPs, we refer to them as PRS with lasso-type winner’s curse correction.

### Simulation results

We performed simulations to evaluate the performance of six PRS prediction methods: 1D and 2D PRS without and with winner’s curse correction (MLE and lasso-type correction). To make simulations realistic in terms of the distribution of minor allele frequencies (MAF) and LD, we simulated quantitative traits with specific genetic architecture by conditioning on the genotypes of a lung cancer GWAS [27], which had 11,924 samples of European ancestry and 485,315 autosomal SNPs after quality control. We randomly selected 10,000 samples as a discovery set and 1,924 as a validation set. The causal SNP set consisted of 5,000 SNPs in linkage equilibrium. In the first set of simulations, the HP SNPs were randomly selected from LD-pruned SNPs across the genome. In the second set of simulations, we simulated HP SNPs located in conserved regions (CR) [28], which were recently reported to be highly enriched for association signal of 17 complex traits based on a heritability partitioning analysis [23].

**Fig 2:**
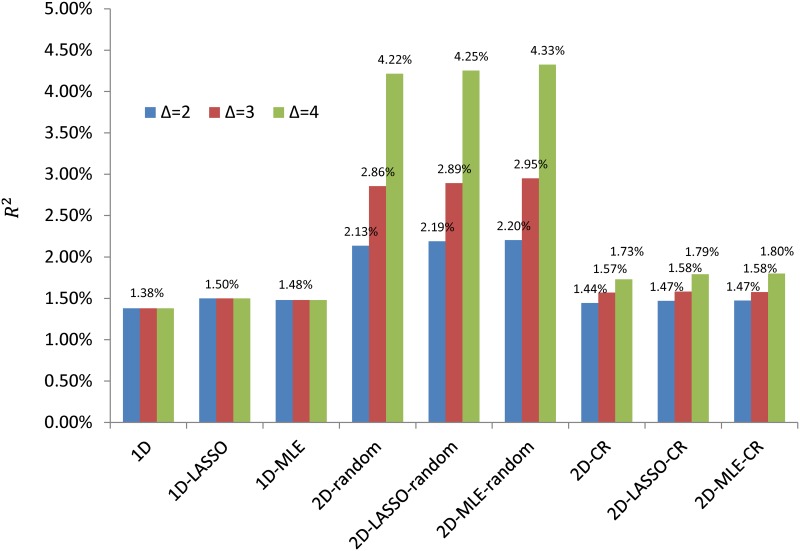
Simulation results for comparing polygenic risk prediction methods and different high priority SNP sets. Quantitative traits were simulated conditioning on the genotypes of LD-pruned SNPs in lung cancer GWAS with 10,000 discovery samples and 1,924 validation samples. For each simulation, we used 5,000 causal SNPs and 9,940 high priority (HP) SNPs (either randomly selected or the SNPs related with conserved regions). Δ denotes the enrichment fold change of the HP SNP. In the x-axis, “1D” denotes 1D PRS without winner’s curse correction; “1D-LASSO(MLE)” denotes 1D PRS with lasso-type (MLE) correction; “2D-random” indicates 2D PRS with HP SNP sets randomly selected from the LD-pruned SNPs in the genome; “2D-CR” indicates 2D PRS using SNPs in conserved regions as HP SNPs.

The simulation results are summarized in Fig 2. First, winner’s curse corrections slightly improved prediction in most if not all simulations and in particular improved more for the 1D PRS than the 2D PRS. We also observed that the two winner’s curse correction methods performed similarly. Second, if HP SNPs were chosen randomly in the LD-pruned SNP set and were strongly enriched for causal SNPs, 2D PRS substantially improved the prediction over 1D PRS. As expected, the improvement increased quickly with the enrichment fold change Δ. Consistent with theoretical analysis assuming independent SNPs (Fig 1B), the optimal *P*-value threshold for HP SNPs was more liberal than that for LP SNPs (S1 Table).

However, when we used CR-SNPs as the HP SNPs, the improvement of 2D PRS was less compared to the simulations with randomly selected HP SNPs, even with the same enrichment fold change. To investigate whether the difference was caused by different local LD structure, for each SNP, we counted the number of SNPs located less than 1Mb from the given SNP and had *r*^2^ ≥ 0.8 with the SNP in The 1000 Genomes Project [29]. For 9,940 CR-SNPs used for our simulations, the average number of LD SNPs is 22.4 (median = 12) while the average number is 6.4 (median = 2) for non-CR SNPs. See also the histograms in S1 Fig. Thus, CR-SNPs are enriched in regions with strong LD and may suggest a possible explanation why CR-SNPs (and other functional categories with similar LD structure) may not lead to improvement in risk prediction as much as would be expected based on enriched heritability.

### Results of analyzing real GWAS data sets

We applied the six PRS methods to 14 traits with either individual level GWAS data or summary level data (Tables 1 and 2). We defined the HP SNP set *S*_1_ using expression QTL SNPs (eSNPs) in blood, tissue specific eSNPs and methylation QTL SNPs (meSNPs), SNPs related with *cis*-regulatory elements (referred to as CRE-SNPs), SNPs related with genomic regions conserved across mammals (referred to as CR-SNPs) and SNPs identified by pleiotropic analyses (referred to as PT-SNPs). Details about annotation data are provided in Materials and Methods. The annotation data used for each trait is summarized in S2 Table. For those with individual level data but without independent validation samples, we used cross-validation to estimate performance.

**Table 1:** GWAS data sets with individual level data.

Data source	Ancestry	Diseases	(Cases, controls)	Cross-validation
WTCCC	European	Bipolar disorder	(1817, 2928)	5-fold
	European	Coronary artery disease	(1878, 2928)	5-fold
	European	Crohn’s disease	(1729, 2928)	5-fold
	European	Hypertension	(1934, 2928)	5-fold
	European	Rheumatoid	(1894, 2928)	5-fold
	European	Type 1 diabetes	(1939, 2928)	5-fold
NCI GWAS	European	Bladder cancer	(5937, 10862)	10-fold
	Asian	Lung cancer in non-smoking females	(5510, 4544)	10-fold
	European	Pancreatic cancer	(5066, 8807)	10-fold

**Table 2:** GWAS data with summary level data.

	Discovery sample			Validation sample		
	Ancestry	Data	(Cases, controls)	Ancestry	Data	(Cases, controls)
Type 2 diabetes	European	DIAGRAM GERA	(17802, 105109)	Europe	GERA	(1500,1500)
Lung cancer	European	TRICL	(11300, 15952)	Europe	PLCO	(1237,1330)
Schizophrenia	European	PGC2	(31560,42951)	Europe	MGS	(2681,2653)
Colorectal cancer	European	GECCO	(9719, 10937)	Europe	PLCO	(1000,2302)
Prostate cancer	Europe African Japanese Latino	PRACTICAL ELLIPSE	(38703, 40796)	Europe	Pegsus	(4600,2941)

#### Polygenic risk prediction of type 2 diabetes

**Fig 3:**
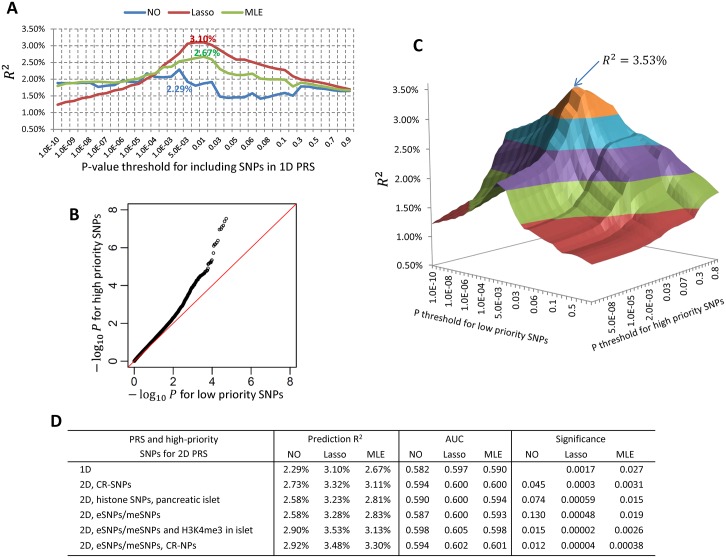
Genetic risk prediction for type-2 diabetes. PRS models were built based on the summary statistics from a meta-analysis of DIAGRAM consortium and GERA data (17,802 cases and 105,109 controls in total) and validated in independent 1500 cases and 1500 controls in GERA. (A) Prediction R^2^ (observational scale) for 1D PRS with or without winner’s curse correction. “NO”: no winner’s correction for association coefficients; “Lasso”: regression coefficients were modified by a lasso-type correction; “MLE”: association coefficients were modified by maximizing a likelihood function conditioning on selection. (B) Quantile-quantile plot for −*log*_10_(*P*) for high priority (HP) SNPs vs. low priority (LP) SNPs. SNPs were pruned to have pairwise *r*^2^ ≤ 0.1. Here, the HP SNPs were eSNPs/meSNPs in adipose tissue or SNPs related with the H3K4me3 mark in pancreatic islet cell line with data downloaded from the ROADMAP project. The HP SNPs were strongly enriched in the discovery data. (C) Prediction R^2^ for 2D PRS with lasso-type winner’s curse correction. The SNP set was the same to (B). The best prediction (R^2^ = 3.53%) was achieved when we included HP SNPs using criterion *P* ≤ 0.03 and LP SNPs with *P* ≤ 0.005. (D) The prediction R^2^, the area under the curve (AUC) and the significances for testing whether an alternative PRS was better than the standard 1D.

We first use type-2 diabetes (T2D) as an example to illustrate our methods. Fig 3A presents the 1D PRS results for T2D. The standard 1D PRS without winner’s curse correction had a prediction R^2^ = 2.29% by including SNPs with P≤2×10^−3^. The winner’s curse correction improved R^2^ to 3.10% using the lasso-type correction and 2.67% using the MLE correction.

Next, we investigated whether functional annotation could further improve risk prediction. We considered CR-SNPs, eSNPs and meSNPs in adipose tissue, and SNPs related with different histone marks and their combinations as HP SNP sets. These SNPs were enriched in T2D GWAS, exemplified by the QQ plot in Fig 3B for a HP SNP set comprising of eSNPs/meSNPs in adipose tissue and SNPs related with H3K4me3 in the pancreatic islet cell line. Note that the SNPs have been pruned to have pairwise *r*^2^ ≤ 0.1, so the observed enrichment was unlikely due to an artifact related to extensive LD. Fig 3C illustrates how the prediction R^2^ of a 2D PRS depends on the *P*-value thresholds for the HP and LP SNPs. The prediction R^2^ was maximized using a more liberal *P*-value threshold 0.03 for HP SNPs and a more rigorous threshold 0.005 for LP SNPs. This optimal 3D PRS had 8,018 HP SNPs and 2,033 LP SNPs.

Fig 3D reports the prediction R^2^, AUC and the significance for testing whether an alternative PRS method could improve the standard 1D PRS. The best predictions were achieved by the 2D PRS with lasso-type correction: R^2^ = 3.48% using eSNPs/meSNPs and CR-SNPs and R^2^ = 3.53% using eSNPs/meSNPs and H3K4me3 SNPs in pancreatic islet cell line (52.0% and 54.1% efficiency gain compared to 2.29% using standard 1D PRS, respectively). These improvements were statistically significant compared to the 1D standard PRS (*P* = 0.00002 and 0.00004, respectively). Of note, the recently developed method LD-pred [31] that models the LD information only slightly improved prediction R^2^ from 2.47% to 2.73% (10.5% efficiency gain) using DIAGRAM summary statistics as discovery. Results are summarized in S3 Table (prediction R^2^, AUC and Nagelkerke R^2^), S4 Table (*P*-value for testing significance of improvement) and S5 Table (optimal thresholds for SNP selection).

#### Results for WTCCC data

**Fig 4:**
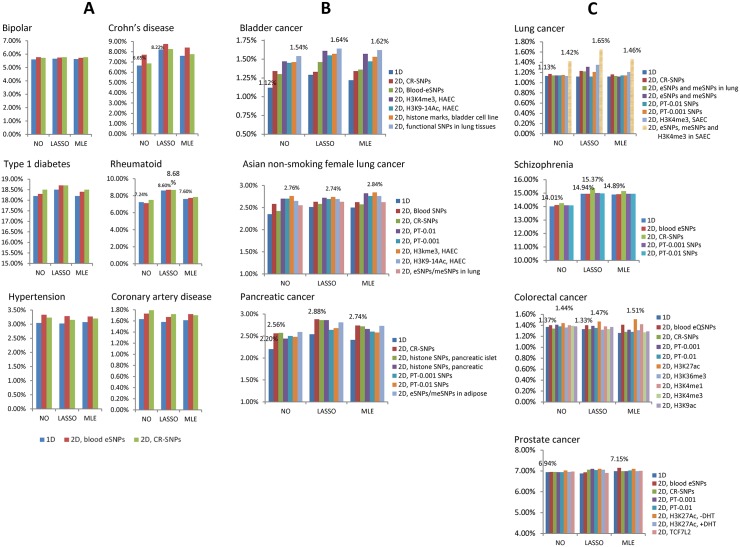
Comparison of polygenic risk prediction methods for 13 complex diseases. For all figures, the y-coordinate is the prediction R^2^ in the observational scale. “1D” denotes 1D PRS; “2D, blood eSNPs” denotes 2D PRS using blood eSNPs as high-prior SNP set. In the x-axis, “NO” denotes PRS without winner’s curse correction; “LASSO” and “MLE” denote lasso-type and MLE winner’s curse correction, respectively. (A) Prediction R^2^ values for six diseases in WTCCC data, estimated based on five-fold cross-validation. (B) Prediction R^2^ values for three GWAS of cancers, estimated based on ten-fold cross-validation. (C) Prediction R^2^ values for four complex diseases estimated based on independent validation samples.

The prediction R^2^ values for six diseases in WTCCC data are reported in Fig 4A. The AUCs and Nagelkerke R^2^ are summarized in S6 Table. Optimal thresholds for SNP selection are in S7 Table. The lasso-type winner’s curse correction improved the 1D PRS predictions for CD, RA and T1D. The 2D PRS improved the prediction for CD (6.65% to 7.71% using blood eSNPs). Combining functional data and lasso-type correction gave a prediction R^2^ = 8.75% for CD (31.6% efficiency gain over the standard 1D PRS). However, because of the small sample size in the validation sample, the improvements were not statistically significant.

#### Results for three cancer GWAS with individual genotype data

Results are summarized in Fig 4B (prediction R^2^), S8 Table (AUC and Nagelkerke R^2^), S9 Table (*P*-value for testing significance of improvement) and S10 Table (optimal thresholds for SNP selection). The standard 1D PRS achieved an R^2^ = 1.12% for bladder cancer, 2.35% for Asian nonsmoking female lung cancer and 2.2% for pancreatic cancer, indicating the difficulty of genetic risk prediction for these cancers. The 2D PRS with lasso-type correction improved the prediction although the various annotation datasets gave different improvement. For bladder cancer, the greatest efficiency gain (R^2^ = 1.64%, 46.4% efficiency gain over the standard 1D PRS and 27.1% efficiency gain over the 1D PRS with lasso-type correction) was achieved with the SNPs related to the lung tissue/cell line expression data (eSNPs, meSNPs, H3K4me3 SNPs in SAEC), which performed slightly better than the SNPs related with histone marks in bladder cell line (R^2^ = 1.46%). For non-smoking female Asian lung cancer, the 2D PRS incorporated with PT-0.001 SNPs or H3K4me3 SNPs in HAEC improved R^2^ to 2.84%. For pancreatic cancer, the 2D PRS incorporated with CR-SNPs, SNPs related with histone marks of pancreatic islet and adipose eSNPs/meSNPs improved prediction R^2^ by approximately ~30% compared with the standard 1D PRS. Many of the improvements over the standard 1D PRS were statistically significant (S9 Table), e.g., P = 0.025 for 2D PRS with H3K4me3 SNPs in HAEC for bladder cancer, P = 0.025 for 2D PRS with PT-0.001 SNPs for Asian lung cancer and P = 0.047 (0.023, 0.023) for 2D PRS with CR-SNPs (PT-0.001, PT-0.01 SNPs) for pancreatic cancer.

#### Results for four large-scale summary-statistics datasets

Prediction results for lung cancer, schizophrenia, prostate cancer and colorectal cancer are reported in Fig 4C (prediction R^2^), S3 Table (AUC and Nagelkerke R^2^), S4 Table (*P*-values for testing whether improvements were significant), S5 Table (optimal p-value thresholds for SNP selection in 2D PRS) and S2 Fig. For lung cancer, the standard 1D PRS had an R^2^ = 1.13%. The best prediction R^2^ = 1.65% was achieved by lasso-corrected 2D PRS with eSNPs/meSNPs in lung tissues, blood eSNPs and SNPs related with H3K4me3 in SAEC. To achieve this prediction accuracy, the optimal *P*-value threshold for the 2D PRS should be 0.008 for HP SNPs and 5 × 10^−6^ for LP SNPs. However, the improvement was not statistically significant. For schizophrenia, the lasso-type correction improved 1D PRS R^2^ from 14.01% to 14.94%; the 2D PRS with CR-SNPs further improved the R^2^ to 15.37% slightly and the improvement was highly statistically significant (P = 3.2 × 10^−10^). For CRC and prostate cancer, neither winner’s curse correction nor 2D PRS improved prediction.

## Discussion

Our study demonstrates that the predictive performance of GWAS PRS models can be improved based on a combination of a simple adjustment to the threshold levels of SNP selection and weights of selected SNPs. The degree of gain, however, is not uniform and depends on multiple factors, including the genetic architecture of the trait, sample size of the discovery sample set, degree of enrichment of association in selected set of “high-prior” SNPs and the linkage disequilibrium patterns of these SNPs with the rest of the genome.

The simple winner’s curse correction of SNP weights using the lasso-type method leads to an improvement in performance of PRS uniformly across all studied diseases. For some diseases, such as type-2 diabetes (Fig 3 and S3 Table) or Crohn’s disease (Fig 4 and S6 Table), this correction alone led to notable improvement in the performance of PRS. The optimal weighting of SNPs would depend on the true effect size distribution of the underlying susceptibility SNPs. Lasso-type weights can be expected to be optimal under a double exponential distribution [19, 32], and it is possible that the weighting could be improved further under alternative models of effect-size distribution. It is, however, encouraging that irrespective of what might be the true effect-size distribution, which is likely to vary across the diseases of study; our simple lasso-type correction improves over the standard PRS without adding any additional computational complexity.

The effect of using various threshold levels for different functional categories of SNPs on the performance of the model varied by disease as well as the functional annotation of external data sets employed in our analytical approach. After adjustment with lasso-type weights, the use of two-dimensional threshold based on prioritized SNPs led to notably higher values of R^2^ for lung cancer in Caucasians, bladder cancer, type-2 diabetes and pancreatic cancer. Consistent with theoretical expectations, for each of the traits, the optimal thresholds selected were more liberal for the associated category of high-prior SNPs than those for complementary set.

Our simulation study illustrated how the improvement in performance of the PRS model due to differential treatment of certain categories of SNPs is modest even when these SNPs have been categorized to be highly enriched for heritability [22]. For example, recent heritability partitioning analysis has identified SNPs in conserved DNA regions, representing 2.6% of the genome, to be highly enriched for GWAS heritability for many diseases (explaining 35% heritability on average). Our theoretical calculations suggest that if only independent SNPs are analyzed, use of a subset of SNPs similarly enriched for heritability is expected to yield much higher improvement in the performance of the model (Fig 1). Our simulation studies showed that a similarly large gain is expected even in the presence of naturally occurring LD pattern if these SNPs are selected randomly from the genome. However, when we simulated high-prior SNPs based on the exact location of conserved regions, the improvement was modest, within the range of observed data. The CR-SNPs represent a highly unusual linkage disequilibrium pattern in that they are in high degree of LD with an unusually large number of neighboring SNPs (S1 Fig).

In the future, more detailed and accurate assessment of the functional annotation of SNPs should improve performance of PRS models. Our method requires only simple modifications to the standard PRS algorithm and can thereby be used to rapidly evaluate the effectiveness of many alternative strategies. In the current study, we used physical location information pertaining to histone marks to define high-priority SNP. However, a SNP located in histone marks does not necessarily cause the variation in histone binding. Thus, a more reasonable approach is to identify genetic variants associated with histone variation across subjects in order to define high-priority SNP sets. These types of histone QTLs have recently been reported in small-scale studies based on HapMap samples [33, 34]. We expect that histone QTL SNPs identified in future large-scale tissue specific studies might be more informative for risk prediction.

We have investigated the performance of the various algorithms using criteria that reflect how much of the variability of the observed outcomes can be explained by the PRS in the validation dataset. For clinical applications of risk-models, however, it is important to evaluate whether models are well calibrated that is to what extent they can produce unbiased estimates of risk for individuals with different SNP profiles. Earlier studies have noted that the standard PRS can be mis-calibrated and additional calibration steps may be needed when applying PRS in a clinical setting. In this regard, we find that a winner’s curse correction can alleviate calibration bias of the standard PRS, but substantial residual bias remains in some situations (S11 Table). The regression relationship between overall PRS and disease status can be estimated based on a relatively small validation sample and can also be used to re-scale PRS for producing calibrated risk estimates.

We used several different metrics for evaluating the potential impact of an improved PRS for risk-stratification. The percentage gain in prediction R^2^ due to improved PRS is substantial for several diseases. For these diseases, the impact of an improved PRS on overall discriminatory performance of the models is noticeable but small (increase in AUC value between 1–2%). However, even a modest increment in AUC value can lead to identification of substantially higher fraction of individuals who are at the tails of risk distribution and hence likely to consider clinical decisions (S12 Table).

A limitation of our method is that we use stringent LD-pruning for creating sets of independent SNPs. However, this may result in loss of predictive power of models as SNPs in moderate or low LD may still harbor independent association signals. The LD-pred [31] method has been proposed to better account for correlated SNPs in building PRS using GWAS summary-level data and has been shown to lead to improved performance over standard PRS for some diseases such as schizophrenia. The LD-pred method also uses a specific form of prior distribution for obtaining “shrunken” estimates of the regression coefficients for the SNPs in the model. Although we did not make direct comparisons, it appears that the LD-pred method gains over standard PRS by improving the accounting for correlation between risk SNPs. In contrast, in our algorithm, which used stringent LD pruning, the gain in performance over the standard PRS mainly came from the lasso-type winner’s curse correction and the use of variable thresholds to account for HP and LP SNPs. Thus it is possible that in the future the complementary strengths of the algorithms can be combined to develop more powerful PRS.

In conclusion, we have proposed a set of simple methods for constructing PRS for genetic risk prediction using GWAS summary-level data. The proposed methods are computationally not onerous and yet show a noteworthy gain in performance. A major strength of our study is that we evaluated the proposed methods across a large number of scenarios reflecting a spectrum of underlying genetic architectures for different complex diseases, sample size of the study and available functional annotation. These studies and additional simulations provide comprehensive insights to promises and limitations of genetic risk prediction models in the near future.

## Materials and Methods

### LD-pruning and LD-clumping

The performance of PRS is typically improved if genetic markers are pruned for LD. LD-pruning procedures that ignore GWAS *P*-values frequently prune out the most significant SNPs and may reduce performance. Instead, we use the LD-clumping procedure implemented in PLINK [20] that chooses the most significant SNP from a set of SNPs in LD guided by GWAS *P*-values. After LD-clumping, no SNPs with physical distance less than 500kb have LD *r*^2^ ≥ 0.1.

### Expanding HP SNP set through LD

Suppose *S*_*1*_ is a given HP set defined based on external annotation data (see section *Annotation datasets*). Any SNP in high LD with a SNP in *S*_*1*_ is also considered to be an HP SNP. Thus, we expanded *S*_*1*_ by including all SNPs that were in high LD (*r*^2^ ≥ 0.8) with any SNP in the original *S*_*1*_.

### Simulations

We simulated quantitative traits with specific genetic architecture by conditioning on the genotypes of a lung cancer GWAS [27], including 11,924 samples of European ancestry and 485,315 autosomal SNPs after quality control. The simulation scheme is summarized in the following steps:

1. We performed LD-pruning implemented in PLINK so that no SNPs within 500kb were in LD at threshold *r*^2^ = 0.1. After LD-pruning, *M* = 53,163 autosomal SNPs (denoted as *S*) were left.
2. Denote *S*_1_ as the putative HP SNP set and *S*_2_ = *S* \ *S*_1_ as the LP SNP set. We selected a set of 5000 “causal” SNPs (denoted as *C*) from the pruned SNP set *S*. If *C* is randomly selected, *i*.*e*., *S*_1_ is not enriched with causal SNPs, we expect | *S*_1_ ∩ *C* | = | *C* || *S*_1_| / *M* SNPs overlapping between *S*_1_ and *C*. Thus, we defined the enrichment fold change for *S*_1_ as
  $$\Delta =\frac{\left|S_{1}\cap C\right|}{|C||S_{1}|/M}\;.$$
  The enrichment fold change Δ ranged from 2 to 4 in simulations.
3. We simulated quantitative traits according to *y*_*i*_ = Σ_*t*∈*C*_ *β*_*t*_*g*_*it*_ + *ε*_*i*_, where *β*_*t*_*s* were simulated independently from a Gaussian mixture distribution $\beta_{t}\sim \pi N(0,\sigma_{1}^{2})+(1-\pi )N(0,\sigma_{2}^{2})$ with *π* = 0.1 Here, $\sigma_{1}^{2}$, $\sigma_{2}^{2}$ and *Var*(*ε*_*i*_) were scaled so that *Var*(*y*_*i*_) = 1. The phenotypic variances explained by the two components were $h_{1}^{2}=|C|\pi \sigma_{1}^{2}=0.1$ and $h_{2}^{2}=|C|(1-\pi )\sigma_{2}^{2}=0.4$. We assume the same effect-size distribution for both HP and LP causal SNPs, but the proportions of causal SNPs are higher in the former than the later group. Under this assumption, Δ also reflects the ratio of heritability explained at a per SNP basis in the HP set compared to LP set.
4. We randomly selected 10,000 samples as a discovery set and 1,924 as a validation set. We performed GWAS association analysis for all 485,315 autosomal SNPs in the discovery sample. The summary statistics were used to calculate PRS for each sample in the validation sample. The prediction *R*^2^ was calculated as max_*λ*_ *cor*^2^(*PRS*_*i*_(*λ*), *y*_*i*_) for 1D PRS methods and max_*λ*_1__,_*λ*_2__ *cor*^2^(*PRS*_*i*_(*λ*_1_, *λ*_2_), *y*_*i*_) for 2D PRS methods. We repeated the simulation 50 times for each set of parameters and report the average prediction *R*^2^.

Recently, Finucane et al. [23] reported the heritability explained by common SNPs in multiple functional categories for 17 traits. Interestingly, they found that common SNPs located in regions that are conserved in mammals [28] accounted for about 2.6% of total common SNPs but explained approximately 35% of total heritability in average across these traits, suggesting a 13.5-fold enrichment. Thus, we were motivated to investigate whether SNPs related with the conserved regions (CR) may be useful for 2D PRS methods. We downloaded the CR annotations (http://compbio.mit.edu/human-constraint/data/gff/), identified common SNPs located in any CR and also identified their LD SNPs with *r*^2^ ≥ 0.8. These SNPs are referred to as CR-SNPs, which were used as HP *S*_1_ in simulations. We found 9,940 CR-SNPs overlapping with the 53,163 LD-pruned SNPs. To investigate whether specific genomic locations of CR-SNPs influence the performance of 2D-PRS, we also performed simulations using a set *S*_1_ of random SNPs that has the same size and associated heritability as the CR-SNPs.

### WTCCC GWAS data

The Wellcome Trust Case Control Consortium [30] (WTCCC) data consisted of two control data sets (1958 Cohort samples and NBS control samples) and seven diseases: bipolar disorder (BD), coronary artery disease (CAD), Crohn’s disease (CD), hypertension (HT), rheumatoid arthritis (RA), Type 1 diabetes (T1D) and Type 2 diabetes (T2D). Since we analyzed T2D using a much larger discovery sample, we did not analyze the T2D data in WTCCC. Because cases and controls were genotyped in different batches, differential errors between cases and controls might cause a serious overestimate of the risk prediction. Thus, we performed very rigorous quality control (QC) by removing duplicate samples, first or second degree relatives, samples with missing rate greater than 5% and non-European samples identified from EigenStrat [35] analysis. For each disease, we excluded SNPs with MAF<5%, missing rate >2%, missing rate difference >1% between cases and controls or *P*_HWE_<10^−4^ in the control samples. For each PRS method and each disease, we estimated the prediction R^2^ by five-fold cross-validation.

### Three cancer GWAS with individual genotype data

We analyzed three cancer GWAS with individual level genotype data: the bladder cancer [36, 37] GWAS of European ancestry including 5,937 cases and 10,862 controls, the pancreatic cancer GWAS [38] of European ancestry (after excluding samples with Asian or African ancestry) including 5,066 cases and 8,807 controls, and the Asian non-smoking female lung cancer GWAS [39] with 5,510 cases and 4,544 controls. After QC, the bladder cancer GWAS had 463,559 autosomal SNPs and the Asian lung cancer GWAS had 329,703 autosomal SNPs. The pancreatic cancer GWAS included samples from three studies that used different genotyping platforms. For convenience, we analyzed 267,935 autosomal SNPs that overlapped in all three platforms. The prediction performance was evaluated using ten-fold cross-validation.

### Five large GWAS with summary statistics and independent validation samples

For T2D, we downloaded the summary statistics of the DIAGRAM (DIAbetes Genetics Replication And Meta-analysis) consortium [40] with 12,171 cases and 56,862 controls for 2.5 million SNPs imputed to the Hapmap2 reference panel. We also downloaded the GERA (Genetic Epidemiology Research on Adult Health and Aging) GWAS data of European ancestry with 7,131 T2D patients and 49,747 samples without T2D (but may have other medical conditions, e.g., 27.4% with cancers, 25.4% with asthma, 25.4% with allergic rhinitis‎ and 12.4% with depression). We randomly selected 5,631 T2D patients and 48,247 non-T2D subjects from GERA as discovery set, performed association analysis adjusting for top 10 PCA scores and meta-analyzed with the summary statistics from DIAGRAM for 353,196 autosomal SNPs overlapping between the two studies. The resulting summary statistics were used to build PRS risk models, which were validated in the remaining 1500 T2D patients and 1500 non-T2D subjects in GERA.

The PGC2 (Psychiatric Genetics Consortium) schizophrenia GWAS meta-analysis consisted of 34,241 cases and 45,604 controls [41] (http://www.med.unc.edu/pgc/downloads). Summary statistics were obtained by meta-analyzing all PGC2 schizophrenia GWAS except the MGS [42] (Molecular Genetics of Schizophrenia) subjects of European ancestry. The summary statistics were used to build PRS models, which were validated in MGS samples with 2,681 cases and 2653 controls.

The TRICL (Transdisciplinary Research in Cancer of the Lung) GWAS consortium consisted of 12,537 lung cancer cases and 17,285 controls [43, 44]. We performed meta-analysis using TRICL samples excluding the samples from the PLCO [27] (Prostate, Lung, Colon, and Ovary Cohort Study) study. The summary statistics based on 11,300 cases and 15,952 controls were used to build risk models, which were validated in the PLCO lung GWAS samples with 1,237 cases and 1,333 controls.

For colorectal cancer, we performed meta-analysis for the GECCO (Genetics and Epidemiology of Colorectal Cancer Consortium) [45] GWAS data after excluding the PLCO GWAS data. The PLCO samples were genotyped using two different genotyping platforms with different marker densities: one had approximately 500K SNPs and the other had only 250K SNPs. Thus, we first imputed the genotypes to the Hapmap2 reference panel using IMPUTE2 [46] and selected SNPs with imputation *r*^2^ ≥ 0.9 for risk prediction. The discovery sample consisted of 9,719 cases and 10,937 controls from 19 studies. The PLCO validation sample had 1,000 cases and 2,302 controls.

The summary statistics for prostate cancer were obtained from the PRACTICAL (PRostate cancer AssoCiation group To Investigate Cancer Associated aLterations) consortium and The GAME-ON/ELLIPSE (Elucidating Loci Involved in Prostate Cancer Susceptibility) Consortium with samples from populations of European, African, Japanese and Latino ancestry [5]. The discovery samples consisted of 38,703 cases and 40,796 controls after excluding the NCI Pegsus GWAS samples with 4,600 cases and 2,941 controls, which were used for validation. We analyzed 536,057 autosomal SNPs after QC that overlapped between the validation and the discovery sample summary statistics.

### Annotation data sets

For many traits, GWAS risk SNPs have been reported to show enrichment for eQTLs, methylation QTLs (meQTLs) and *cis*-regulatory elements (CREs). In addition, recent studies have reported extensive genetic pleiotropy across diseases and traits, e.g. psychiatric diseases [47, 48], schizophrenia and cardiovascular-disease risk factors, including blood pressure, triglycerides, low- and high-density lipoprotein, body mass index (BMI) and waist-to-hip ratio (WHR) [49]. This information may potentially improve risk prediction if the SNPs identified from the secondary trait are highly enriched in the GWAS of the primary trait. Thus, we defined the HP SNP set *S*_1_ using eQTL SNPs (referred to as eSNPs) in blood, tissue specific eSNPs and meQTL SNPs (referred to as meSNPs), SNPs related with CREs (referred to as CRE-SNPs), SNPs related with genomic regions conserved across mammals (referred to as CR-SNPs) and SNPs identified by pleiotropic analyses (referred to as PT-SNPs). Here, LD was calculated based on the genotype data of relevant ancestry in The 1000 Genomes Project [29]. Note that the availability of functional annotation data depends on tissue types. However, for all diseases studied in the paper, we have used blood eSNPs and CR-SNPs because blood eSNPs are enriched for GWAS of all these traits and CR-SNPs were highly enriched in many traits by a heritability partitioning analysis [23].

#### eSNPs and meSNPs

Blood *cis*-eSNPs were identified from two large-scale eQTL studies in European populations. One study involved a transcriptome sequencing project of 922 subjects [50] and the other involved a microarray study of 5,311 subjects [51] (http://genenetwork.nl/bloodeqtlbrowser/). Because of its very large sample size, the second study had the power to detect eSNPs with even tiny effect sizes which may not have meaningful functional importance. Thus, we included eSNPs with association *P*-value <10^−6^ with any gene in the *cis* region in the second study. For both Asian and European lung cancer GWAS data, we used eSNPs [52] and meSNPs [53] based on lung tissues. For T2D, we used eSNPs [54] and meSNPs [55] based on adipose tissues (http://www.muther.ac.uk/Data.html). Furthermore, detected *trans*-SNPs are much fewer than *cis*-SNPs and the replication rate of *trans*-eSNPs was much lower than *cis*-SNPs [54], suggesting that including *trans*-SNPs would be unlikely to improve risk prediction. Thus, we did not include *trans*-SNPs.

#### CRE-SNPs

CREs are regions of noncoding DNA regulating the transcription of nearby genes. SNPs located in CREs may change the binding of specific transcription factors and thus the expression of the target genes. Typically, CREs are identified through ChIP-Seq experiments of histone modifications. We downloaded “peak” data (each peak represents one CRE) of specific sets of histone methylation markings, acetylation markings and DNase I hypersensitive sites (DHSs) from the ROADMAP project website for relevant cell lines. For each identified CRE (‘peak’), we identified common SNPs with MAF>1%. For prostate cancer, we used the ChIP-Seq data for H3K27Ac and the transcription factor TCF7L2 [56] to define HP SNP sets.

#### PT-SNPs

The summary statistics for height [1, 2], BMI and obesity [3, 57], WHR [58], waist circumference (WC) [58], hip circumference (HIP) [58] were downloaded from the GIANT consortium website. The summary statistics for GWAS meta-analysis of cardiovascular-disease risk factors [59], including triglycerides (TG), low-density lipoprotein (LDL) and high-density lipoprotein (HDL), were also used for 2D PRS.

We investigated whether or not each tentative HP SNP set was enriched for GWAS associations by examining the quantile-quantile (QQ) plot, which was made for HP SNPs vs. LP SNPs after LD-clumping. The SNP sets not enriched for GWAS associations were not expected to improve risk prediction in 2D PRS. Thus, for each disease, we only included HP SNP sets for 2D PRS when they showed strong enrichment in QQ plots. Interestingly, blood eSNPs were enriched for almost all diseases. CR-SNPs showed modest enrichment for majority of the diseases. Thus, blood eSNPs and CR-SNPs were used for 2D PRS for all diseases. In addition, eSNPs and meSNPs derived in lung tissues were enriched in lung cancer GWAS of both European and Asian ancestry. The SNPs related in enhancer and active promoter regions (characterized by H3K4me3, H3K9-14Ac, H3K36me3, H3K4me1, H3K9ac and H3K9me3) were enriched for GWAS associations but SNPs related with the repressive regions (characterized by H3K27me3) were not. Thus, we included SNPs related with these enhancer and active promoter regions for 2D PRS. DHS SNPs were not strongly enriched and thus were excluded. Recently, we have shown significantly shared genetic component between lung cancer and bladder cancer risk [60]. Thus, we also used HP SNPs derived based on lung tissues or cell lines for predicting bladder cancer risk. Furthermore, we found that SNPs identified through pleiotropic analysis were enriched in multiple diseases. For example, SNPs with *P*-value <0.001 in GWAS of height, HDL, LDL, TC, TG, WC, obesity, HIP and T2D were enriched in lung cancer GWAS. Because our 2D PRS methods required a relatively large number of HP SNPs to achieve improvement, we combined the SNPs with *P*-value <10^−3^ (or 10^−2^) in at least one trait into a HP SNP set referred as PT-0.001 (or PT-0.01).

### Testing the statistical significance of improvement for risk prediction

For WTCCC and three cancer GWAS data sets with individual genotype data, we used *K*-fold cross-validation to estimate prediction R^2^. Here, *K* = 5 for WTCCC data and *K* = 10 for cancer GWAS data. We were interested in testing whether the prediction of a new PRS method was significantly better than that of the standard 1D PRS defined in Eq (1). For the *i*^*th*^ cross-validation, we denote $R_{i,0}^{2}$ as the maximum prediction for the standard 1D PRS optimized across *P*-value thresholds, $R_{i,1}^{2}$ as the maximum prediction for a new PRS method optimized across all *P*-value thresholds for 1D PRS and all pairs of *P*-value thresholds for 2D PRS. We defined $\delta_{i}=\;R_{i,1}^{2}\;-R_{i,0}^{2}$ and estimated its variance as ${\hat{\sigma }}^{2}=\Sigma_{i=1}^{K}{\left(\delta_{i}-\bar{\delta }\right)}^{2}/\left(K-1\right)$ with $\bar{\delta }=\Sigma_{i=1}^{K}\delta_{i}/K$. We calculated the statistic $T=\bar{\delta }/\sqrt{{\hat{\sigma }}^{2}/K}$ and evaluated its significance using the *t*-distribution. For the five diseases with independent validation samples, we used bootstrap to estimate the variance of the R^2^ estimates to test significance [29].

### Theoretical prediction performance assuming independent SNPs

Suppose that for a given trait of interest *Y*, there are two predefined SNP sets: the high priority (HP) SNP set *S*_1_ and the low priority (LP) SNP set *S*_2_. SNPs have been pruned and are in linkage equilibrium. We assume that *S*_1_ has *M*_1_ independent susceptibility SNPs and *M*_3_ null SNPs while *S*_2_ has *M*_2_ susceptibility SNPs and *M*_4_ independent null SNPs. Following Chatterjee et al. [11], we assume that the true relationship between outcome *Y* and independent susceptibility SNPs is modeled as follows:

$$Y=\overset{M_{1}}{\underset{i=1}{\sum }}\beta_{1i}g_{1i}+\;\overset{M_{2}}{\underset{j=1}{\sum }}\beta_{2j}g_{2j}+\overset{M_{3}}{\underset{k=1}{\sum }}0\cdot g_{3k}+\overset{M_{4}}{\underset{l=1}{\sum }}0\cdot g_{4l}+\epsilon ,$$

where all *Y* and the genotypic values *g*’s are standardized so that *E*(*Y*) = 0, *Var*(*Y*) = 1, *E*(*g*) = 0 and *Var*(*g*) = 1, and the error term *ϵ* ~ *N*(0, *σ*^2^) and is independent of the genotypic values.

From a discovery GWAS data set of size *N*, we have regression coefficient ${\hat{\beta }}_{i}$- and two-sided p-value *P*_*i*_ for each SNP. We build an additive prediction model by including SNPs in *S*_1_ with *P*-value ≤ *α*_1_ and SNPs in *S*_2_ with *P*-value ≤ *α*_2_:

$$\hat{Y}\left(\alpha_{1},\alpha_{2}\right)=\overset{M_{1}}{\underset{i=1}{\sum }}{\hat{\beta }}_{1i}\gamma_{1i}\left(\alpha_{1}\right)g_{1i}+\;\overset{M_{2}}{\underset{j=1}{\sum }}{\hat{\beta }}_{2j}\gamma_{2j}\left(\alpha_{2}\right)g_{2j}+\overset{M_{3}}{\underset{k=1}{\sum }}{\hat{\beta }}_{3k}\gamma_{3k}\left(\alpha_{1}\right)g_{3k}+\overset{M_{4}}{\underset{l=1}{\sum }}{\hat{\beta }}_{4l}\gamma_{4l}\left(\alpha_{2}\right)g_{4l},$$

where *γ* (*α*) = *I* (*P* ≤ *α*) with *I* (⋅) being an indicator function.

The predictive correlation coefficient (PCC) for the predictive model can be expressed as

$$\begin{array}{l} PCC\left(\alpha_{1},\alpha_{2}\right)=cor\left(Y,\hat{Y}\left(\alpha_{1},\alpha_{2}\right)\right)\\ =\frac{\sum_{i=1}^{M_{1}}\beta_{1i}{\hat{\beta }}_{1i}\gamma_{1i}\left(\alpha_{1}\right)+\;\sum_{j=1}^{M_{2}}\beta_{2j}{\hat{\beta }}_{2j}\gamma_{2j}\left(\alpha_{2}\right)}{\sqrt{\sum_{i=1}^{M_{1}}{\hat{\beta }}_{1i}^{2}\gamma_{1i}\left(\alpha_{1}\right)+\;\sum_{j=1}^{M_{2}}{\hat{\beta }}_{2j}^{2}\gamma_{2j}\left(\alpha_{2}\right)+\sum_{k=1}^{M_{3}}{\hat{\beta }}_{3k}^{2}\gamma_{3k}\left(\alpha_{1}\right)+\sum_{l=1}^{M_{4}}{\hat{\beta }}_{4l}^{2}\gamma_{4l}\left(\alpha_{2}\right)}}. \end{array}$$

Following Chatterjee et al. (2014), one can verify that PCC follows a normal distribution by the central limit theorem and the strong law of large numbers. Therefore, the expected value of PCC can be approximated as

$$\begin{array}{l} E\left(PCC\left(\alpha_{1},\alpha_{2}\right)\right)=\frac{\sum_{i=1}^{M_{1}}\beta_{1i}e_{N,\alpha_{1}}\left(\beta_{1i}\right)\text{pow}\left(N,\beta_{1i},\alpha_{1}\right)+\;\sum_{j=1}^{M_{2}}\beta_{2j}e_{N,\alpha_{2}}\left(\beta_{2j}\right)\text{pow}\left(N,\beta_{2j},\alpha_{2}\right)}{\sqrt{\sum_{i=1}^{M_{1}}\nu_{N,\alpha_{1}}\left(\beta_{1i}\right)\text{pow}\left(N,\beta_{1i},\alpha_{1}\right)+\sum_{j=1}^{M_{2}}\nu_{N,\alpha_{2}}\left(\beta_{2j}\right)\text{pow}\left(N,\beta_{2j},\alpha_{2}\right)+M_{3}\alpha_{1}\nu_{N,\alpha_{1}}\left(0\right)+M_{4}\alpha_{2}\nu_{N,\alpha_{2}}\left(0\right)}}\;,\\ \approx \frac{M_{1}\int \beta e_{N,\alpha_{1}}\left(\beta \right)\text{pow}\left(N,\beta ,\alpha_{1}\right)f_{1}\left(\beta \right)d\beta +\;M_{2}\int \beta e_{N,\alpha_{2}}\left(\beta \right)\text{pow}\left(N,\beta ,\alpha_{2}\right)f_{2}\left(\beta \right)d\beta }{\sqrt{M_{1}\int \beta \nu_{N,\alpha_{1}}\left(\beta \right)\text{pow}\left(N,\beta ,\alpha_{1}\right)f_{1}\left(\beta \right)d\beta +M_{2}\int \beta \nu_{N,\alpha_{2}}\left(\beta \right)\text{pow}\left(N,\beta ,\alpha_{2}\right)f_{2}\left(\beta \right)d\beta +M_{3}\alpha_{1}\nu_{N,\alpha_{1}}\left(0\right)+M_{4}\alpha_{2}\nu_{N,\alpha_{2}}\left(0\right)}} \end{array}$$

where $e_{N,\alpha }\left(\beta \right)=E\left(\hat{\beta }|\gamma \left(\alpha \right)=1\right)$, $\nu_{N,\alpha }\left(\beta \right)=E\left({\hat{\beta }}^{2}|\gamma \left(\alpha \right)=1\right)$, pow (*N*, *β*, *α*) is power to detect a SNP with effect size *β* at a significance level *α* in a GWAS with size *N*, and *f*_1_(⋅) and *f*_2_(⋅) are effect-size distributions for HP and LP susceptibility SNPs, respectively.

In our numerical calculations, we assumed that the effect sizes of the susceptibility SNPs in the HP and LP sets followed the same distribution $\beta \sim \pi N\left(0,\sigma_{1}^{2}\right)+\left(1-\pi \right)N\left(0,\sigma_{2}^{2}\right)$, consistent with simulations. We performed grid search to identify the p-value thresholds (*α*_1_, *α*_2_) that maximizes *E*(*PCC*(*α*_1_, *α*_2_)). For binary disease outcomes, AUC can be expressed as a function of PCC [11].

## Supporting Information

S1 Table Optimal P-value thresholds for including SNPs for 1D and 2D PRS in simulation studies. (DOC)

S2 Table GWAS and functional annotation data for developing genetic risk prediction models. (DOC)

S3 Table Prediction R^2^, Nagelkerke R^2^ and AUC for five large scale GWAS summary statistics with independent validation data. (DOC)

S4 Table P-values for testing whether a PRS statistically significantly improved the risk prediction for five large-scale GWAS summary statistics based on bootstrap. (DOC)

S5 Table Optimal P-value thresholds for including SNPs for 1D and 2D PRS for five diseases with large-scale discovery data and independent validation samples. (DOC)

S6 Table Prediction R^2^, Nagelkerke R^2^ and AUC in WTCCC, based on five-fold cross-validation. (DOC)

S7 Table Optimal P-value thresholds for including SNPs for 1D and 2D PRS for WTCCC data. (DOC)

S8 Table Prediction R^2^, Nagelkerke R^2^ and AUC in the three cancer GWAS data sets, based on 10-fold cross-validation. (DOC)

S9 Table P-values for testing whether a PRS significantly improved the risk prediction for three cancer GWAS. (DOC)

S10 Table Optimal P-value thresholds for including SNPs for 1D and 2D PRS for three cancer GWAS. (DOC)

S11 Table Calibration comparison for 1D PRS modeling with or without winner’s curse correction. (DOC)

S12 Table Implication of identifying high-risk subjects based on PRS. (DOCX)

S1 Text Additional acknowledegements. (DOC)

S1 Fig Randomly selected SNPs and SNPs related with conserved genomic regions (CR-SNPs) have different local linkage disequilibrium (LD) pattern. (TIF)

S2 Fig The prediction R2 for four diseases with large-scale discovery samples. (TIF)
