# Supplementary material for: Winner's Curse Correction and Variable Thresholding Improve Performance of Polygenic Risk Modeling Based on Genome-Wide Association Study Summary-Level Data
Source: PLoS Genet. 2016 Dec 30;12(12):e1006493. doi: 10.1371/journal.pgen.1006493 (PMC5201242; doi:10.1371/journal.pgen.1006493)
Supplement: S1 Table — (DOC) [file pgen.1006493.s001.doc]

**S1 Table:** **Optimal P-value thresholds for including SNPs for 1D and 2D PRS in simulation studies.**

|  | 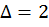 | 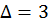 | 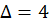 |
| --- | --- | --- | --- |
| 1D | 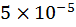 | 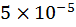 | 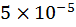 |
| 1D-LASSO | 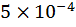 | 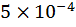 | 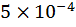 |
| 1D- MLE | 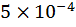 | 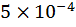 | 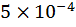 |
| 2D-random | (0.01,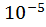) | (0.04,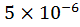) | (0.08,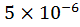) |
| 2D-random-LASSO | (0.03,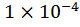) | (0.08,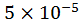) | (0.2,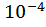) |
| 2D-random-MLE | (0.04,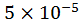) | (0.08,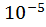) | (0.2,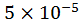) |
| 2D-CR | (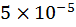,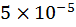) | (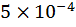,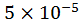) | (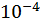,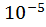) |
| 2D-CR-LASSO | (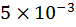,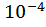) | (0.002,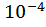) | (0.001,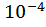) |
| 2D-CR-MLE | (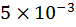,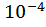) | (0.002,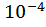) | (0.001,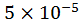) |

For each parameter setting, 50 simulations were performed and the P-value thresholds reported in the tables are the median of the 50 simulations. This table corresponds to the results reported in Figure 4. For 2D PRS, the two P-value thresholds correspond to the high-priority SNP set and the low priority SNP set.

“1D” denotes 1D PRS without winner’s curse correction; “1D-LASSO(MLE)” denotes 1D PRS with LASSO-type (MLE) correction; “2D-random” indicates 2D PRS with functional SNP sets randomly selected from the LD-pruned SNPs in the genome; “2D-CR” indicates 2D PRS using SNPs in conserved regions as functional SNPs.
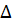
is the enrichment fold change for the high-priority SNPs.
