# Supplementary material for: Winner's Curse Correction and Variable Thresholding Improve Performance of Polygenic Risk Modeling Based on Genome-Wide Association Study Summary-Level Data
Source: PLoS Genet. 2016 Dec 30;12(12):e1006493. doi: 10.1371/journal.pgen.1006493 (PMC5201242; doi:10.1371/journal.pgen.1006493)
Supplement: S2 Table — (DOC) [file pgen.1006493.s002.doc]

**S2 Table: GWAS data for developing genetic risk prediction models.**

**(A): Disease GWAS with individual genotype data used for evaluating risk prediction performance.**

| Data source | Diseases | Ancestry | (Cases, controls) | Cross-validation | Functional SNP sets for 2D PRS |
| --- | --- | --- | --- | --- | --- |
| WTCCC | Bipolar disorder | Europe | (1817, 2928) | 5-fold | blood eSNPs^1,2^, CR-SNPs^3^ |
|  | Coronary artery disease | Europe | (1878, 2928) | 5-fold |  |
|  | Crohn’s disease | Europe | (1729, 2928) | 5-fold |  |
|  | Hypertension | Europe | (1934, 2928) | 5-fold |  |
|  | Rheumatoid | Europe | (1894, 2928) | 5-fold |  |
|  | Type 1 diabetes | Europe | (1939, 2928) | 5-fold |  |
|  |  |  |  |  |  |
| Three cancer GWAS with individual genotype data | Bladder cancer | Europe | (5937, 10862) | 10-fold | blood eSNPs^1,2^, CR-SNPs^3^, active histone marks H3K4me3 and H3K9-14Ac in HAEC^4^, active histone marks in bladder cell lines downloaded from the ROADMAP project, lung related functional SNPs (eSNPs^5^ and meSNPs^6^ in lung tissues, H3K4me and H3K-14Ac in HAEC^4^) |
|  | Lung cancer,  Asian non-smoking females | Asian | (5510, 4544) | 10-fold | blood eSNPs^1,2^, CR-SNPs^3^, eSNPs^5^ and meSNPs^6^ in lung tissues, active histone marks H3K4me3 and H3K9-14Ac in HAEC^4^, pleiotropic SNPs with p<0.01 (denoted as PT-0.01) or p<0.001 (denoted as PT-0.001) in at least one other trait. |
|  | Pancreatic cancer | Europe | (5066, 8807) | 10-fold | CR-SNPs^3^, eSNPs/meSNPs in adipose^7,8^, combined active histone mark (H3K4me3, H3K9-14Ac, H3K36me3, H3K4me1, H3K9ac and H3K9me3) SNPs in pancreatic islet cells and primary pancreatic cells downloaded from the ROADMAP project, PT-0.01 and PT-0.001 SNPs. |

**(B): Disease GWAS with independent validation samples for evaluating prediction performance.**

|  |  | Discovery sample | | Validation sample | |  |
| --- | --- | --- | --- | --- | --- | --- |
|  | Ancestry | Data sources | (Cases, controls) | Data sources | (Cases, controls) | Functional SNP sets for 2D PRS |
| Type 2 diabetes | Europe | DIAGRAM  GERA | (17,802, 105,109) | GERA | (1500,1500) | CR-SNPs^3^, eSNPs/meSNPs in adipose^7,8^, histone mark SNPs in pancreatic islet cells. |
| Lung cancer | Europe | TRICL | (11,300, 15,952) | PLCO | (1237,1330) | blood eSNPs^1,2^, eSNPs^5^ and meSNPs^6^ in lung tissues, CR-SNPs^3^, H3K4me3 in SAEC^9^, PT-0.01 and PT-0.001 SNPs. |
| Schizophrenia | Europe | PGC2 | (31,560,42,951) | MGS | (2681,2653) | blood eSNPs^1,2^, CR-SNPs^3^, PT-0.01 and PT-0.001 SNPs |
| Colorectal cancer | Europe | GECCO | (9,719, 10,937) | PLCO | (1000,2302) | blood eSNPs^1,2^, CR-SNPs^3^, PT-0.01 and PT-0.001 SNPs, histone mark SNPs in colon/rectal cells in the ROADMAP project . |
| Prostate cancer | Europe  African  Japanese  Latino | PRACTICAL  ELLIPSE | (38,703, 40,796) | Pegsus | (4600,2941) | blood eSNPs^1,2^, CR-SNPs^3^, PT-0.01 and PT-0.001 SNPs, TCF7L2/H3K27Ac (-DHT)/H3K27Ac(+DHT) in LNCaP cells^10^. |

1. Battle, A. *et al.* Characterizing the genetic basis of transcriptome diversity through RNA-sequencing of 922 individuals. *Genome Research* **24**, 14-24 (2014).

2. Westra, H.J. *et al.* Systematic identification of trans eQTLs as putative drivers of known disease associations. *Nature Genetics* **45**, 1238-U195 (2013).

3. Lindblad-Toh, K. *et al.* A high-resolution map of human evolutionary constraint using 29 mammals. *Nature* **478**, 476-482 (2011).

4. Marconett, C., Zhou, B., Rieger, M., Selamat, S. & Mickael Dubourd, X.F., Sean K. Lynch, Kimberly D. Siegmund, Benjamin P. Berman, Zea Borok, Ite A. Laird-Offringa. Integrated transcriptomic and epigenomic analysis reveals novel pathways regulating distal lung epithelial cell differentiation. *PlosGenet* (2013).

5. Hao, K. *et al.* Lung eQTLs to Help Reveal the Molecular Underpinnings of Asthma. *Plos Genetics* **8**(2012).

6. Shi, J. *et al.* Characterizing the genetic basis of methylome diversity in histologically normal human lung tissue. *Nat Commun* **5**, 3365 (2014).

7. Grundberg, E. *et al.* Mapping cis- and trans-regulatory effects across multiple tissues in twins. *Nat Genet* **44**, 1084-9 (2012).

8. Grundberg, E. *et al.* Global analysis of DNA methylation variation in adipose tissue from twins reveals links to disease-associated variants in distal regulatory elements. *Am J Hum Genet* **93**, 876-90 (2013).

9. Consortium, T.E.P. A user's guide to the encyclopedia of DNA elements (ENCODE). *PLoS Biol* **9**(2011).

10. Hazelett, D.J. *et al.* Comprehensive Functional Annotation of 77 Prostate Cancer Risk Loci. *Plos Genetics* **10**(2014).
