# Supplementary material for: Winner's Curse Correction and Variable Thresholding Improve Performance of Polygenic Risk Modeling Based on Genome-Wide Association Study Summary-Level Data
Source: PLoS Genet. 2016 Dec 30;12(12):e1006493. doi: 10.1371/journal.pgen.1006493 (PMC5201242; doi:10.1371/journal.pgen.1006493)
Supplement: S3 Table — (DOC) [file pgen.1006493.s003.doc]

**S3 Table: Prediction R^2^ (=cor(y,PRS)^2^), Nagelkerke R^2^ and AUC for five large scale GWAS summary statistics with independent validation data.**

| Disease | PRS and high-priority  SNPs for 2D PRS | Prediction R^2^ | | | Nagelkerke R^2^ | | | AUC | | |
| --- | --- | --- | --- | --- | --- | --- | --- | --- | --- | --- |
|  |  | Winner’s curse correction | | | Winner’s curse correction | | | Winner’s curse correction | | |
|  |  | NO | LASSO | MLE | NO | LASSO | MLE | NO | LASSO | MLE |
| T2D | 1D | **2.29%** | 3.10% | 2.67% | **3.05%** | 4.13% | 3.56% | **0.582** | 0.597 | 0.590 |
|  | 2D, CR-SNPs | 2.73% | 3.32% | 3.11% | 3.64% | 4.43% | 4.15% | 0.594 | 0.600 | 0.600 |
|  | 2D, histone SNPs, pancreatic islet | 2.58% | 3.23% | 2.81% | 3.44% | 4.32% | 3.75% | 0.590 | 0.600 | 0.594 |
|  | 2D, eSNPs/meSNPs | 2.58% | 3.28% | 2.83% | 3.44% | 4.38% | 3.78% | 0.587 | 0.600 | 0.593 |
|  | 2D, eSNPs/meSNPs and H3K4me3 in islet | 2.90% | **3.53%** | 3.13% | 3.87% | **4.71%** | 4.17% | 0.598 | **0.605** | 0.598 |
|  | 2D, eSNPs/meSNPs, CR-NPs | 2.92% | 3.48% | 3.30% | 3.89% | 4.65% | 4.41% | 0.594 | 0.602 | 0.601 |
| EUR lung | 1D | **1.13%** | 1.12% | 1.12% | **1.52%** | 1.48% | 1.50% | **0.564** | 0.563 | 0.563 |
|  | 2D, CR-SNPs | 1.17% | 1.23% | 1.16% | 1.55% | 1.64% | 1.55% | 0.564 | 0.564 | 0.564 |
|  | 2D, eSNPs and meSNPs in lung | 1.14% | 1.22% | 1.13% | 1.52% | 1.63% | 1.51% | 0.563 | 0.566 | 0.563 |
|  | 2D, eSNPs and meSNPs | 1.14% | 1.31% | 1.12% | 1.52% | 1.75% | 1.49% | 0.564 | 0.571 | 0.563 |
|  | 2D, PT-0.01 SNPs | 1.14% | 1.12% | 1.14% | 1.52% | 1.49% | 1.52% | 0.564 | 0.563 | 0.563 |
|  | 2D, PT-0.001 SNPs | 1.15% | 1.21% | 1.14% | 1.54% | 1.61% | 1.52% | 0.567 | 0.567 | 0.563 |
|  | 2D, H3K4me3, SAEC | 1.13% | 1.35% | 1.21% | 1.51% | 1.80% | 1.61% | 0.560 | 0.569 | 0.565 |
|  | 2D, eSNPs, meSNPs and H3K4me3 in SAEC | 1.14% | **1.65%** | 1.25% | 1.52% | **1.98%** | 1.67% | 0.566 | **0.574** | 0.567 |
| Prostate | 1D | 6.94% | 6.87% | 6.98% | 9.43% | 9.35% | 9.48% | 0.654 | 0.652 | 0.654 |
|  | 2D, blood eSNPs | 6.95% | 6.93% | 7.15% | 9.44% | 9.43% | 9.72% | 0.654 | 0.653 | 0.656 |
|  | 2D, CR-SNPs | 6.95% | 7.05% | 6.98% | 9.44% | 9.58% | 9.49% | 0.654 | 0.653 | 0.654 |
|  | 2D, PT-0.001 | 6.94% | 7.10% | 6.98% | 9.43% | 9.67% | 9.49% | 0.654 | 0.655 | 0.654 |
|  | 2D, PT-0.01 | 6.94% | 7.04% | 7.02% | 9.43% | 9.58% | 9.55% | 0.654 | 0.654 | 0.654 |
|  | 2D, H3K27Ac, -DHT | 7.02% | 7.10% | 7.10% | 9.54% | 9.65% | 9.65% | 0.655 | 0.655 | 0.655 |
|  | 2D, H3K27Ac, +DHT | 6.95% | 7.06% | 6.98% | 9.45% | 9.60% | 9.48% | 0.654 | 0.654 | 0.653 |
|  | 2D, TCF7L2 | 6.96% | 6.90% | 7.00% | 9.45% | 9.38% | 9.51% | 0.654 | 0.652 | 0.654 |

| Disease | PRS and high-priority  SNPs for 2D PRS | Prediction R^2^ | | | Nagelkerke R^2^ | | | AUC | | |
| --- | --- | --- | --- | --- | --- | --- | --- | --- | --- | --- |
|  |  | Winner’s curse correction | | | Winner’s curse correction | | | Winner’s curse correction | | |
|  |  | NO | LASSO | MLE | NO | LASSO | MLE | NO | LASSO | MLE |
| CRC | 1D | 1.37% | 1.33% | 1.26% | 1.93% | 1.87% | 1.78% | 0.571 | 0.570 | 0.568 |
|  | 2D, blood eSNPs | 1.40% | 1.40% | 1.41% | 1.97% | 1.96% | 1.98% | 0.570 | 0.571 | 0.572 |
|  | 2D, CR-SNPs | 1.34% | 1.33% | 1.28% | 1.92% | 1.86% | 1.78% | 0.570 | 0.570 | 0.568 |
|  | 2D, PT-0.001 | 1.41% | 1.39% | 1.32% | 1.93% | 1.92% | 1.81% | 0.570 | 0.571 | 0.569 |
|  | 2D, PT-0.01 | 1.38% | 1.35% | 1.28% | 1.97% | 1.93% | 1.84% | 0.571 | 0.571 | 0.570 |
|  | 2D, H3K27ac | 1.44% | 1.47% | 1.51% | 2.04% | 2.07% | 2.11% | 0.571 | 0.570 | 0.571 |
|  | 2D, H3K36me3 | 1.36% | 1.32% | 1.31% | 1.93% | 1.86% | 1.84% | 0.571 | 0.570 | 0.569 |
|  | 2D, H3K4me1 | 1.40% | 1.38% | 1.42% | 1.98% | 1.95% | 2.00% | 0.571 | 0.571 | 0.570 |
|  | 2D, H3K4me3 | 1.39% | 1.33% | 1.27% | 1.96% | 1.88% | 1.80% | 0.572 | 0.570 | 0.569 |
|  | 2D, H3K9ac | 1.38% | 1.37% | 1.29% | 1.96% | 1.92% | 1.82% | 0.571 | 0.571 | 0.569 |
| SCZ | 1D | **14.01%** | 14.94% | 14.89% | **18.75%** | 19.99% | 19.91% | **0.717** | 0.724 | 0.724 |
|  | 2D, blood eSNPs | 14.10% | 14.94% | 14.91% | 18.88% | 19.99% | 19.94% | 0.718 | 0.724 | 0.723 |
|  | 2D, CR-SNPs | 14.25% | **15.37%** | 15.15% | 19.03% | **20.56%** | 20.24% | 0.718 | **0.727** | 0.725 |
|  | 2D, PT-0.001 SNPs | 14.09% | 15.00% | 14.95% | 18.83% | 20.02% | 20.00% | 0.717 | 0.724 | 0.724 |
|  | 2D, PT-0.01 SNPs | 14.07% | 14.97% | 14.95% | 18.85% | 19.99% | 19.95% | 0.718 | 0.724 | 0.724 |
