# Supplementary material for: Winner's Curse Correction and Variable Thresholding Improve Performance of Polygenic Risk Modeling Based on Genome-Wide Association Study Summary-Level Data
Source: PLoS Genet. 2016 Dec 30;12(12):e1006493. doi: 10.1371/journal.pgen.1006493 (PMC5201242; doi:10.1371/journal.pgen.1006493)
Supplement: S4 Table — (DOC) [file pgen.1006493.s004.doc]

**S4** Table: P-values for testing whether a PRS statistically significantly improved the risk prediction for five large-scale GWAS summary statistics based on bootstrap.

| Disease | PRS and high-priority  SNPs for 2D PRS | Winner’s curse correction | | |
| --- | --- | --- | --- | --- |
|  |  | NO | LASSO | MLE |
| CRC | 1D |  | 0.5879 | 0.7412 |
|  | 2D, blood eSNPs | 0.4152 | 0.4404 | 0.4245 |
|  | 2D, CR-SNPs | 0.6179 | 0.5755 | 0.6736 |
|  | 2D, PT-0.001 | 0.3040 | 0.4621 | 0.5941 |
|  | 2D, PT-0.01 | 0.4638 | 0.5442 | 0.6736 |
|  | 2D, H3K27ac | 0.3632 | 0.3556 | 0.2951 |
|  | 2D, H3K36me3 | 0.5362 | 0.6038 | 0.6239 |
|  | 2D, H3K4me1 | 0.4207 | 0.4790 | 0.3962 |
|  | 2D, H3K4me3 | 0.4207 | 0.5793 | 0.7007 |
|  | 2D, H3K9ac | 0.4715 | 0.5000 | 0.6631 |
| SCZ | 1D |  | 1.5E-11 | 2.2E-09 |
|  | 2D, blood eSNPs | 1.7E-01 | 1.5E-11 | 6.0E-08 |
|  | 2D, CR-SNPs | 2.0E-01 | 3.2E-10 | 5.8E-06 |
|  | 2D, PT-0.001 SNPs | 3.5E-02 | 3.1E-10 | 1.6E-08 |
|  | 2D, PT-0.01 SNPs | 2.7E-01 | 9.9E-10 | 8.8E-08 |
| T2D | 1D |  | 0.00173 | 0.02748 |
|  | 2D, CR-SNPs | 0.04529 | 0.00030 | 0.00313 |
|  | 2D, histone SNPs, pancreatic islet | 0.07353 | 0.00059 | 0.01513 |
|  | 2D, eSNPs/meSNPs | 0.13234 | 0.00048 | 0.01890 |
|  | 2D, eSNPs/meSNPs and H3K4me3 in islet | 0.01468 | 0.00002 | 0.00256 |
|  | 2D, eSNPs/meSNPs, CR-NPs | 0.01222 | 0.00004 | 0.00038 |
| EUR lung | 1D |  | 0.5285 | 0.5662 |
|  | 2D, CR-SNPs | 0.4166 | 0.3446 | 0.4300 |
|  | 2D, eSNPs and meSNPs in lung | 0.4778 | 0.3478 | 0.5000 |
|  | 2D, eSNPs and meSNPs | 0.4778 | 0.2169 | 0.5199 |
|  | 2D, PT-0.01 SNPs | 0.4693 | 0.5222 | 0.4668 |
|  | 2D, PL-0.001 SNPs | 0.4532 | 0.3085 | 0.4715 |
|  | 2D, H3K4me3, SAEC | 0.5000 | 0.1694 | 0.3581 |
|  | 2D, eSNPs, meSNPs and H3K4me3 in SAEC | 0.4878 | 0.1399 | 0.3413 |
| Prostate | 1D |  | 0.6306 | 0.2866 |
|  | 2D, blood eSNPs | 0.4602 | 0.5173 | 0.0401 |
|  | 2D, CR-SNPs | 0.4327 | 0.3162 | 0.3581 |
|  | 2D, PT-0.001 | 0.5000 | 0.2767 | 0.3792 |
|  | 2D, PT-0.01 | 0.5000 | 0.3446 | 0.2692 |
|  | 2D, H3K27Ac, -DHT | 0.2119 | 0.2611 | 0.1587 |
|  | 2D, H3K27Ac, +DHT | 0.4594 | 0.3222 | 0.4070 |
|  | 2D, TCF7L2 | 0.3170 | 0.5721 | 0.2209 |
