# Supplementary material for: Winner's Curse Correction and Variable Thresholding Improve Performance of Polygenic Risk Modeling Based on Genome-Wide Association Study Summary-Level Data
Source: PLoS Genet. 2016 Dec 30;12(12):e1006493. doi: 10.1371/journal.pgen.1006493 (PMC5201242; doi:10.1371/journal.pgen.1006493)
Supplement: S5 Table — (DOC) [file pgen.1006493.s005.doc]

**S5 Table: Optimal P-value thresholds for including SNPs for 1D and 2D PRS for five diseases with large-scale discovery data and independent validation samples.**

| Disease | PRS and high-priority  SNPs for 2D PRS | P-value thresholds | | |
| --- | --- | --- | --- | --- |
|  |  | Winner’s curse correction | | |
|  |  | NO | LASSO | MLE |
| T2D | 1D | 0.002 | 0.008 | 0.01 |
|  | 2D, CR-SNPs | (0.002, 5×10^-8^) | (0.02,0.005) | (0.01, 0.00005) |
|  | 2D, histone SNPs, pancreatic islet | (0.1,0.002) | (0.03,0.008) | (0.02,0.005) |
|  | 2D, eSNPs/meSNPs | (0.02,0.002) | (0.03,0.008) | (0.02,0.002) |
|  | 2D, eSNPs/meSNPs and H3K4me3 in islet | (0.01, 5×10^-5^) | (0.03,0.005) | (0.02, 0.002) |
|  | 2D, eSNPs/meSNPs, CR-NPs | (0.01, 5×10^-5^) | (0.02,0.005) | (0.01,0.0001) |
| EUR lung | 1D | 5×10^-9^ | 10^-4^ | 10^-7^ |
|  | 2D, CR-SNPs | (10^-10^, 5×10^-9^) | (0.02, 5×10^-4^) | (10^-10^, 10^-6^) |
|  | 2D, eSNPs and meSNPs in lung | (5×10^-9^, 10^-10^) | (10^-4^, 5×10^-10^) | (10^-7^, 10^-10^) |
|  | 2D, eSNPs and meSNPs | (5×10^-9^, 10^-10^) | (0.01, 5×10^-6^) | (10^-7^, 10^-10^) |
|  | 2D, PT-0.01 SNPs | (10^-10^, 5×10^-9^) | (5×10^-5^,5×10^-6^) | (5×10^-8^, 10^-6^) |
|  | 2D, PL-0.001 SNPs | (0.001, 5×10^-9^) | (0.002,5×10^-6^) | (5×10^-8^, 10^-6^) |
|  | 2D, H3K4me3, SAEC | (0.002, 5×10^-9^) | (0.008, 10^-5^) | (0.005, 10^-7^) |
|  | 2D, eSNPs, meSNPs and H3K4me3 in SAEC | (0.001, 10^-6^) | (0.008,5×10^-6^) | (0.005,5×10^-6^) |
| Prostate | 1D | 5×10^-6^ | 0.002 | 5×10^-5^ |
|  | 2D, blood eSNPs | (5×10^-6^,5×10^-6^) | (0.03,0.005) | (5×10^-5^,0.001) |
|  | 2D, CR-SNPs | (5×10^-6^, 10^-5^) | (0.02,0.001) | (5×10^-5^, 10^-5^) |
|  | 2D, PT-0.001 | (5×10^-6^,5×10^-6^) | (0.06,0.002) | (5×10^-5^, 10^-5^) |
|  | 2D, PT-0.01 | (5×10^-6^,5×10^-6^) | (0.02,0.002) | (5×10^-5^, 10^-5^) |
|  | 2D, H3K27Ac, -DHT | (5×10^-4^,5×10^-6^) | (0.07,0.002) | (0.04, 5×10^-5^) |
|  | 2D, H3K27Ac, +DHT | (10^-6^,5×10^-6^) | (0.08,0.005) | (0.04, 5×10^-5^) |
|  | 2D, TCF7L2 | (0.005,5×10^-6^) | (0.002,0.02) | (0.005, 5×10^-5^) |
| CRC | 1D | 0.005 | 0.008 | 0.008 |
|  | 2D, blood eSNPs | (0.008,0.005) | (0.008,0.02) | (0.008,0.03) |
|  | 2D, CR-SNPs | (0.008,0.005) | (0.01,0.008) | (0.008,0.005) |
|  | 2D, PT-0.001 | (0.008,0.005) | (0.02,0.008) | (0.03,0.008) |
|  | 2D, PT-0.01 | (0.005,0.005) | (0.01,0.008) | (0.03,0.008) |
|  | 2D, H3K27ac | (0.03,0.005) | (0.04,0.008) | (0.03,0.005) |
|  | 2D, H3K36me3 | (0.008,0.005) | (0.01,0.008) | (0.03,0.008) |
|  | 2D, H3K4me1 | (0.005,0.005) | (0.01,0.008) | (0.03,0.008) |
|  | 2D, H3K4me3 | (0.002,0.005) | (0.008,0.008) | (0.008,0.005) |
|  | 2D, H3K9ac | (0.005,0.005) | (0.01,0.008) | (0.01,0.008) |
| SCZ | 1D | 0.2 | 0.3 | 0.2 |
|  | 2D, blood eSNPs | (0.04,0.2) | (0.3,0.3) | (0.09,0.2) |
|  | 2D, CR-SNPs | (0.5,0.05) | (0.8,0.3) | (0.5,0.1) |
|  | 2D, PT-0.001 SNPs | (0.4,0.2) | (0.3,0.7) | (0.9,0.3) |
|  | 2D, PT-0.01 SNPs | (0.01,0.2) | (0.3,0.3) | (0.07,0.2) |
