# Supplementary material for: Winner's Curse Correction and Variable Thresholding Improve Performance of Polygenic Risk Modeling Based on Genome-Wide Association Study Summary-Level Data
Source: PLoS Genet. 2016 Dec 30;12(12):e1006493. doi: 10.1371/journal.pgen.1006493 (PMC5201242; doi:10.1371/journal.pgen.1006493)
Supplement: S6 Table — (DOC) [file pgen.1006493.s006.doc]

**S6 Table: Prediction R^2^ (=cor(y,PRS)^2^), Nagelkerke R^2^ and AUC in the WTCCC data, based on five-fold cross-validation.**

| Disease | PRS | High priority SNPs for 2D PRS | Prediction R^2^ | | | Nagelkerke R^2^ | | | AUC | | |
| --- | --- | --- | --- | --- | --- | --- | --- | --- | --- | --- | --- |
|  |  |  | Winner’s curse correction | | | Winner’s curse correction | | | Winner’s curse correction | | |
|  |  |  | NO | LASSO | MLE | NO | LASSO | MLE | NO | LASSO | MLE |
| Bipolar disorder | 1D |  | 5.59% | 5.64% | 5.62% | 7.59% | 7.65% | 7.62% | 0.635 | 0.636 | 0.635 |
|  | 2D | Blood eSNPs  CR SNPs | 5.75% | 5.74% | 5.72% | 7.80% | 7.79% | 7.76% | 0.637 | 0.636 | 0.636 |
|  |  |  | 5.72% | 5.75% | 5.76% | 7.76% | 7.80% | 7.81% | 0.637 | 0.637 | 0.637 |
| Coronary artery disease | 1D |  | 1.63% | 1.58% | 1.61% | 2.22% | 2.14% | 2.18% | 0.572 | 0.571 | 0.572 |
|  | 2D | Blood eSNPs  CR SNPs | 1.73% | 1.67% | 1.72% | 2.34% | 2.26% | 2.34% | 0.575 | 0.574 | 0.575 |
|  |  |  | 1.79% | 1.72% | 1.70% | 2.43% | 2.33% | 2.31% | 0.578 | 0.574 | 0.572 |
| Crohn’s disease | 1D |  | **6.65%** | 8.22% | 7.60% | **9.25%** | 11.32% | 10.43% | **0.646** | 0.660 | 0.656 |
|  | 2D | Blood eSNPs  CR SNPs | 7.71% | **8.75%** | 8.40% | 10.59% | **12.10%** | 11.55% | 0.658 | **0.667** | 0.663 |
|  |  |  | 6.85% | 8.25% | 7.75% | 9.34% | 11.40% | 10.61% | 0.651 | 0.660 | 0.656 |
| Hypertension | 1D |  | 3.04% | 3.02% | 3.07% | 4.12% | 4.09% | 4.16% | 0.597 | 0.597 | 0.598 |
|  | 2D | Blood eSNPs  CR SNPs | 3.33% | 3.28% | 3.27% | 4.52% | 4.45% | 4.44% | 0.601 | 0.600 | 0.600 |
|  |  |  | 3.23% | 3.15% | 3.2 | 4.38% | 4.27% | 4.34% | 0.600 | 0.600 | 0.600 |
| Rheumatoid | 1D |  | **7.24%** | 8.60% | 7.60% | **9.77%** | 11.59% | 10.30% | **0.653** | 0.669 | 0.659 |
|  | 2D | Blood eSNPs  CR SNPs | 7.12% | **8.69%** | 7.74% | 9.63% | **11.71%** | 10.44% | 0.650 | **0.671** | 0.658 |
|  |  |  | 7.50% | 8.68% | 7.84% | 10.11% | 11.69% | 10.56% | 0.657 | 0.670 | 0.661 |
| Type 1 diabetes | 1D |  | 18.20% | 18.50% | 18.20% | 26.09% | 26.99% | 26.03% | 0.754 | 0.758 | 0.754 |
|  | 2D | Blood eSNPs  CR SNPs | 18.30% | 18.70% | 18.40% | 26.31% | 26.87% | 26.54% | 0.755 | 0.758 | 0.756 |
|  |  |  | 18.50% | 18.70% | 18.50% | 26.70% | 27.20% | 26.64% | 0.757 | 0.758 | 0.756 |
