# Supplementary material for: Winner's Curse Correction and Variable Thresholding Improve Performance of Polygenic Risk Modeling Based on Genome-Wide Association Study Summary-Level Data
Source: PLoS Genet. 2016 Dec 30;12(12):e1006493. doi: 10.1371/journal.pgen.1006493 (PMC5201242; doi:10.1371/journal.pgen.1006493)
Supplement: S7 Table — (DOC) [file pgen.1006493.s007.doc]

**S7 Table: Optimal P-value thresholds for including SNPs for 1D and 2D PRS for WTCCC data.**

This table corresponds to the results reported in Figure 3 and Supplemental Table S2. For each disease, we have performed five-fold cross-validation. For each cross-validation, we determined the optimal threshold for 1D PRS and a pair of thresholds for 2D PRS. The reported data were the median of the five cross-validation results.

| Disease | PRS | High priority SNPs for 2D PRS |  | | |
| --- | --- | --- | --- | --- | --- |
|  |  |  | Winner’s curse correction | | |
|  |  |  | NO | LASSO | MLE |
| Bipolar disorder | 1D |  | 0.4 | 0.5 | 0.3 |
|  | 2D | Blood eSNPs | (0.3,0.4) | (0.6,0.5) | (0.3,0.4) |
|  |  | CR SNPs | (0.3,0.1) | (0.4,0.2) | (0.9,0.2) |
| Coronary artery disease | 1D |  | 0.6 | 0.7 | 0.6 |
|  | 2D | Blood eSNPs | (0.5,0.4) | (0.8,0.4) | (0.8,0.5) |
|  |  | CR SNPs | (0.5, 0.01) | (0.8,0.2) | (0.7,0.3) |
| Crohn’s disease | 1D |  | 0.0001 | 0.005 | 0.005 |
|  | 2D | Blood eSNPs | (0.001,0.0001) | (0.005,0.005) | (0.005, 0.0005) |
|  |  | CR SNPs | (0.0001,0.00005) | (0.005,0.005) | (0.001, 0.0005) |
| Hypertension | 1D |  | 0.3 | 0.4 | 0.4 |
|  | 2D | Blood eSNPs | (0.4,28) | (0.9,0.3) | (0.6,0.3) |
|  |  | CR SNPs | (0.2,0.3) | (0.4,0.5) | (0.4,0.3) |
| Rheumatoid | 1D |  | 0.000001 | 0.001 | 0.00005 |
|  | 2D | Blood eSNPs | (0.00005,0.00005) | (0.005,0.001) | (0.0001,0.00005) |
|  |  | CR SNPs | (0.00001, 0.00001) | (0.001,0.005) | (0.000000005,0.00005) |
| Type 1 diabetes | 1D |  | 0.00001 | 0.005 | 0.0001 |
|  | 2D | Blood eSNPs | (0.00001,0.000001) | (0.01,0.005) | (0.0005,0.0001) |
|  |  | CR SNPs | (0.000001,0.00005) | (0.001,0.005) | (0.00001,0.00005) |
