# Supplementary material for: Winner's Curse Correction and Variable Thresholding Improve Performance of Polygenic Risk Modeling Based on Genome-Wide Association Study Summary-Level Data
Source: PLoS Genet. 2016 Dec 30;12(12):e1006493. doi: 10.1371/journal.pgen.1006493 (PMC5201242; doi:10.1371/journal.pgen.1006493)
Supplement: S8 Table — (DOC) [file pgen.1006493.s008.doc]

**S8 Table: Prediction R^2^ (=cor(y,PRS)^2^), Nagelkerke R^2^ and AUC in the three cancer GWAS data sets, based on 10-fold cross-validation.**

| Disease | PRS and high-priority  SNPs for 2D PRS | Prediction R^2^ | | | Nagelkerke R^2^ | | | AUC | | |
| --- | --- | --- | --- | --- | --- | --- | --- | --- | --- | --- |
|  |  | Winner’s curse correction | | | Winner’s curse correction | | | Winner’s curse correction | | |
|  |  | NO | LASSO | MLE | NO | LASSO | MLE | NO | LASSO | MLE |
| Pancreatic cancer | 1D | **2.20%** | 2.54% | 2.41% | **3.26%** | 3.68% | 3.47% | 0.587 | 0.594 | 0.590 |
|  | 2D, CR-SNPs | 2.56% | 2.88% | 2.74% | 3.81% | 4.24% | 4.07% | 0.596 | 0.601 | 0.598 |
|  | 2D, histone SNPs, pancreatic islet | 2.57% | **2.86%** | 2.72% | 3.79% | **4.18%** | 3.99% | 0.597 | **0.601** | 0.598 |
|  | 2D, histone SNPs, pancreatic | 2.44% | **2.86%** | 2.66% | 3.57% | **4.18%** | 3.88% | 0.592 | **0.600** | 0.597 |
|  | 2D, PT-0.001 SNPs | 2.50% | 2.64% | 2.60% | 3.63% | 3.83% | 3.75% | 0.594 | 0.597 | 0.595 |
|  | 2D, PT-0.01 SNPs | 2.48% | 2.68% | 2.58% | 3.61% | 3.89% | 3.77% | 0.593 | 0.596 | 0.595 |
|  | 2D, eSNPs/meSNPs in adipose | 2.59% | 2.81% | 2.73% | 3.75% | 4.06% | 3.95% | 0.593 | 0.599 | 0.598 |
| Asian lung | 1D | **2.35%** | 2.51% | 2.50% | **3.15%** | 3.36% | 3.35% | **0.586** | 0.591 | 0.590 |
|  | 2D, blood SNPs | 2.58% | 2.63% | 2.62% | 3.46% | 3.51% | 3.50% | 0.592 | 0.593 | 0.592 |
|  | 2D, CR-SNPs | 2.42% | 2.58% | 2.57% | 3.24% | 3.46% | 3.43% | 0.588 | 0.591 | 0.591 |
|  | 2D, PT-0.01 | 2.70% | 2.72% | 2.82% | 3.61% | 3.64% | 3.77% | 0.593 | 0.594 | 0.595 |
|  | 2D, PT-0.001 | 2.70% | 2.69% | 2.76% | 3.61% | 3.60% | 3.69% | 0.594 | 0.594 | 0.595 |
|  | 2D, H3kme3, HAEC | 2.76% | 2.74% | **2.84%** | 4.09% | 4.07% | **4.20%** | 0.595 | 0.595 | **0.596** |
|  | 2D, H3K9-14Ac, HAEC | 2.65% | 2.69% | 2.76% | 4.02% | 4.07% | 4.15% | 0.593 | 0.594 | 0.596 |
|  | 2D, eSNPs and meSNPs in lung | 2.55% | 2.63% | 2.62% | 4.01% | 3.98% | 4.08% | 0.591 | 0.592 | 0.592 |
| Bladder | 1D | **1.12%** | 1.29% | 1.22% | **1.53%** | 1.78% | 1.68% | **0.561** | 0.565 | 0.563 |
|  | 2D, CR-SNPs | 1.34% | 1.33% | 1.34% | 1.84% | 1.83% | 1.84% | 0.568 | 0.566 | 0.566 |
|  | 2D, blood eSNPs | 1.30% | 1.46% | 1.36% | 1.79% | 2.00% | 1.87% | 0.566 | 0.569 | 0.568 |
|  | 2D, H3K4me3, HAEC | 1.47% | 1.61% | 1.57% | 2.03% | 2.21% | 2.17% | 0.570 | 0.574 | 0.573 |
|  | 2D, H3K9-14Ac, HAEC | 1.45% | 1.55% | 1.47% | 1.99% | 2.13% | 2.02% | 0.570 | 0.573 | 0.571 |
|  | 2D, histone SNPs, OADMAP bladder | 1.46% | 1.57% | 1.53% | 2.01% | 2.15% | 2.11% | 0.571 | 0.572 | 0.571 |
|  | 2D, functional SNPs in lung tissues | 1.54% | **1.64%** | 1.62% | 2.13% | **2.25%** | 2.23% | 0.572 | **0.575** | 0.573 |
