# Supplementary material for: Winner's Curse Correction and Variable Thresholding Improve Performance of Polygenic Risk Modeling Based on Genome-Wide Association Study Summary-Level Data
Source: PLoS Genet. 2016 Dec 30;12(12):e1006493. doi: 10.1371/journal.pgen.1006493 (PMC5201242; doi:10.1371/journal.pgen.1006493)
Supplement: S9 Table — (DOC) [file pgen.1006493.s009.doc]

**S9 Table: P-values for testing whether a PRS statistically significantly improved the risk prediction for three cancer GWAS. P-values were calculated based on a t-statistic with standard deviation estimated by 10-fold cross-validation.**

| Disease | PRS and high-prority  SNPs for 2D PRS | Prediction R^2^ | | |
| --- | --- | --- | --- | --- |
|  |  | Winner’s curse correction | | |
|  |  | NO | LASSO | MLE |
| Pancreatic cancer | 1D |  | 0.176 | 0.104 |
|  | 2D, CR-SNPs | 0.176 | 0.107 | 0.145 |
|  | 2D, histone SNPs, pancreatic islet | 0.109 | 0.059 | 0.071 |
|  | 2D, histone SNPs, pancreatic | 0.149 | 0.063 | 0.055 |
|  | 2D, PT-0.001 SNPs | 0.076 | 0.125 | 0.023 |
|  | 2D, PT-0.01 SNPs | 0.044 | 0.131 | 0.023 |
|  | 2D, eSNPs/meSNPs in adipose | 0.132 | 0.144 | 0.135 |
| Asian lung | 1D |  | 0.220 | 0.124 |
|  | 2D, blood SNPs | 0.113 | 0.111 | 0.058 |
|  | 2D, CR-SNPs | 0.250 | 0.140 | 0.052 |
|  | 2D, PT-0.01 | 0.112 | 0.092 | 0.028 |
|  | 2D, PT-0.001 | 0.078 | 0.082 | 0.025 |
|  | 2D, H3kme3, HAEC | 0.155 | 0.114 | 0.097 |
|  | 2D, H3K9-14Ac, HAEC | 0.200 | 0.221 | 0.137 |
|  | 2D, eSNPs and meSNPs in lung | 0.220 | 0.121 | 0.072 |
| Bladder cancer | 1D | 0.50 | 0.15 | 0.13 |
|  | 2D, CR-SNPs | 0.06 | 0.06 | 0.02 |
|  | 2D, blood eSNPs | 0.15 | 0.12 | 0.13 |
|  | 2D, H3K4me3, HAEC | 0.08 | 0.02 | 0.05 |
|  | 2D, H3K9-14Ac, HAEC | 0.09 | 0.07 | 0.10 |
|  | 2D, histone SNPs, OADMAP bladder | 0.15 | 0.12 | 0.15 |
|  | 2D, functional SNPs in lung tissues | 0.13 | 0.07 | 0.11 |
