# Supplementary material for: Winner's Curse Correction and Variable Thresholding Improve Performance of Polygenic Risk Modeling Based on Genome-Wide Association Study Summary-Level Data
Source: PLoS Genet. 2016 Dec 30;12(12):e1006493. doi: 10.1371/journal.pgen.1006493 (PMC5201242; doi:10.1371/journal.pgen.1006493)
Supplement: S10 Table — (DOC) [file pgen.1006493.s010.doc]

**S10 Table: Optimal P-value thresholds for including SNPs for 1D and 2D PRS for three cancers GWAS.**

This table corresponds to the results reported in Fig 3 and S4 Table. For each disease, we have performed 10-fold cross-validation. For each cross-validation, we determined the optimal threshold for 1D PRS and a pair of thresholds for 2D PRS. The reported data were the median of the ten cross-validation results.

| Disease | PRS and high-priority  SNPs for 2D PRS |  | | |
| --- | --- | --- | --- | --- |
|  |  | Winner’s curse correction | | |
|  |  | NO | LASSO | MLE |
| Pancreatic cancer | 1D | 5×10^-6^ | 10^-3^ | 10^-4^ |
|  | 2D, CR-SNPs | (10^-6^,5×10^-6^) | (0.001, 5×10^-4^) | (5×10^-4^, 10^-5^) |
|  | 2D, histone SNPs, pancreatic islet | (10^-5^,5×10^-6^) | (0.002, 10^-4^) | (5×10^-4^,5×10^-6^) |
|  | 2D, histone SNPs, pancreatic | (5×10^-5^, 10^-5^) | (0.005, 5×10^-5^) | (10^-3^, 5×10^-5^) |
|  | 2D, PT-0.001 SNPs | (10^-5^, 10^-6^) | (10^-3^, 10^-4^) | (5×10^-4^, 10^-5^) |
|  | 2D, PT-0.01 SNPs | (5×10^-6^,5×10^-6^) | (10^-3^, 5×10^-4^) | (5×10^-5^, 10^-5^) |
|  | 2D, eSNPs/meSNPs in adipose | (10^-5^, 5×10^-7^) | (0.002, 10^-4^) | (10^-4^,5×10^-6^) |
| Asian lung | 1D | 5×10^-6^ | 10^-4^ | 10^-5^ |
|  | 2D, blood SNPs | (5×10^-6^, 5×10^-7^) | (5×10^-4^, 10^-4^) | (10^-5^, 10^-5^) |
|  | 2D, CR-SNPs | (7,5×10^-6^) | (5×10^-5^, 10^-4^) | (10^-5^, 10^-5^) |
|  | 2D, PT-0.01 | (10^-5^, 5×10^-7^) | (5×10^-4^, 5×10^-5^) | (10^-4^, 10^-5^) |
|  | 2D, PT-0.001 | (10^-5^, 10^-7^) | (10^-3^, 5×10^-5^) | (10^-4^, 10^-5^) |
|  | 2D, H3kme3, HAEC | (10^-6^, 10^-6^) | (5×10^-4^, 5×10^-5^) | (5×10^-6^, 10^-5^) |
|  | 2D, H3K9-14Ac, HAEC | (1,5×10^-6^) | (10^-5^, 10^-4^) | (5×10^-5^, 5×10^-5^) |
|  | 2D, eSNPs and meSNPs in lung | (10^-6^, 5×10^-7^) | (5×10^-4^, 10^-4^) | (5×10^-6^, 10^-5^) |
| Bladder | 1D | 5×10^-6^ | 10^-4^ | 5×10^-5^ |
|  | 2D, CR-SNPs | (5×10^-6^, 10^-7^) | (5×10^-4^, 10^-4^) | (5×10^-5^, 10^-5^) |
|  | 2D, blood eSNPs | (5×10^-6^,5×10^-6^) | (10^-3^, 10^-4^) | (10^-4^, 0.00005) |
|  | 2D, H3K4me3, HAEC | (10^-4^,5×10^-6^) | (0.005, 10^-4^) | (0.002, 10^-5^) |
|  | 2D, H3K9-14Ac, HAEC | (10^-5^, 5×10^-7^) | (0.005, 10^-4^) | (10^-3^,5×10^-6^) |
|  | 2D, histone SNPs, ROADMAP bladder | (10^-3^, 10^-5^) | (0.005, 5×10^-4^) | (5×10^-4^, 10^-4^) |
|  | 2D, functional SNPs in lung tissues | (10^-4^, 10^-6^) | (0.002, 10^-4^) | (5×10^-4^, 10^-5^) |
