# Supplementary material for: Winner's Curse Correction and Variable Thresholding Improve Performance of Polygenic Risk Modeling Based on Genome-Wide Association Study Summary-Level Data
Source: PLoS Genet. 2016 Dec 30;12(12):e1006493. doi: 10.1371/journal.pgen.1006493 (PMC5201242; doi:10.1371/journal.pgen.1006493)
Supplement: S11 Table — (DOC) [file pgen.1006493.s011.doc]

**S11 Table: Calibration comparison for 1D PRS modeling with or without winner’s curse correction.**

A: WTCCC data. Reported values are based on the average of five-fold cross validation.

| Winner’s curse correction | BD | CAD | CD | HT | RA | T1D | T2D |
| --- | --- | --- | --- | --- | --- | --- | --- |
| NO | 0.043 | 0.021 | 0.364 | 0.036 | 0.710 | 0.649 | 0.303 |
| LASSO | 0.071 | 0.026 | 1.111 | 0.052 | 1.204 | 0.867 | 1.255 |
| MLE | 0.051 | 0.024 | 0.495 | 0.043 | 0.905 | 0.637 | 0.355 |

B: For pancreatic cancer, Asian nonsmoking female lung cancer and bladder cancer, the reported values are based on the average of 10 fold cross validation. For other five diseases, the values are based on independent validation samples.

| Winner’s curse correction | Pancreatic cancer | Bladder cancer | Lung cancer, Asian | Lung cancer, EUR | T2D | Schizophrenia | Prostate cancer | Colorectal cancer |
| --- | --- | --- | --- | --- | --- | --- | --- | --- |
| NO | 0.67 | 0.75 | 0.91 | 0.66 | 0.22 | 0.17 | 0.60 | 0.17 |
| LASSO | 1.83 | 1.82 | 1.88 | 0.96 | 0.74 | 0.31 | 0.86 | 0.86 |
| MLE | 0.69 | 0.66 | 0.99 | 0.67 | 0.26 | 0.23 | 0.61 | 0.28 |

Reported values are the coefficient of the PRS in the logistic regression. A value close to one represents a well-calibrated prediction model. The calibration results for 2D PRS are similar to 1D PRS and are not reported here. LASSO-type winner’s correction has the smallest bias overall.
