# Supplementary material for: Winner's Curse Correction and Variable Thresholding Improve Performance of Polygenic Risk Modeling Based on Genome-Wide Association Study Summary-Level Data
Source: PLoS Genet. 2016 Dec 30;12(12):e1006493. doi: 10.1371/journal.pgen.1006493 (PMC5201242; doi:10.1371/journal.pgen.1006493)
Supplement: S12 Table — (DOCX) [file pgen.1006493.s012.docx]

**S12 Table: Implication of identifying high-risk subjects based on PRS.**

**Type-2 diabetes**

| k | Number of samples (out of 1500 validation samples) with k-fold of population-average risk | | | | Ratio (best-PRS/standard-PRS) |
| --- | --- | --- | --- | --- | --- |
|  | Standard 1D PRS | | Best PRS | | theoretical calculation |
|  | Theoretical calculation | Empirical calculation | Theoretical calculation | Empirical calculation |  |
| 2 | 10.4 | 10 | 30.4 | 31 | 2.93 |
| 3 | 0.1 | 0 | 1.3 | 2 | 12.52 |
| 4 | 0.0014 | 0 | 0.069 | 0 | 51.26 |
| 5 | 0.000026 | 0 | 0.005 | 0 | 190.61 |

Here, we calculate the proportion of samples in the general population identified as high-risk based on a given PRS distribution. For a given PRS, we assume that the PRS risk scores follows a centered normal distribution, i.e. $s\sim N(0,\sigma^{2})$, with parameters estimated based on validation sample. We first perform calibration by fitting a logistic regression $logit\left( y | s \right)=\alpha+\beta s$ to derive $\hat{\beta}$. The calibrated risk score is then $exp(\hat{\beta}s)$. The average risk in the population is then $A=\int\exp\left( \hat{\beta}s \right)\phi(s;0,\sigma)\mathrm{ds}$. To identify samples with projected risk greater than k-fold of the population average risk, we need to find a cut off $s_{0}$ s.t. $\exp\left( \hat{\beta}s_{0} \right)=kA$, i.e., $s_{0}=\log\left( kA \right)/\hat{\beta}$. The theoretical proportion of samples is then calculated by $P(s\geq s_{0})$ assuming a normal distribution $N(0,\sigma^{2})$.

Here, we use T2D to illustrate the calculation. The parameter $\sigma$ was estimated based on the control samples in the validation sample. We calculated the number of samples with k-fold greater risk out of the validation samples using the above theoretical calculations. We also empirically calculate this number based on the PRS in the validation sample. For each disease, we compared our best PRS with the standard 1D PRS without winner’s curse correction or integrating functional data. For T2D, the best PRS is the 2D PRS with eSNPs/meSNPs and H3K4me3 SNPs in pancreatic islet cell line.
