# Supplementary material for: Winner's Curse Correction and Variable Thresholding Improve Performance of Polygenic Risk Modeling Based on Genome-Wide Association Study Summary-Level Data
Source: PLoS Genet. 2016 Dec 30;12(12):e1006493. doi: 10.1371/journal.pgen.1006493 (PMC5201242; doi:10.1371/journal.pgen.1006493)
Supplement: S1 Text — (DOC) [file pgen.1006493.s013.doc]

**Additional acknowledgements**

**Funding for GECOO (Genetics and Epidemiology of Colorectal Cancer) Consortium**

GECCO: National Cancer Institute, National Institutes of Health, U.S. Department of Health and Human Services (U01 CA137088; R01 CA059045). ASTERISK: a Hospital Clinical Research Program (PHRC) and supported by the Regional Council of Pays de la Loire, the Groupement des Entreprises Françaises dans la Lutte contre le Cancer (GEFLUC), the Association Anne de Bretagne Génétique and the Ligue Régionale Contre le Cancer (LRCC). COLO2&3: National Institutes of Health (R01 CA60987). DACHS: German Research Council (Deutsche Forschungsgemeinschaft, BR 1704/6-1, BR 1704/6-3, BR 1704/6-4 and CH 117/1-1), and the German Federal Ministry of Education and Research (01KH0404 and 01ER0814). DALS: National Institutes of Health (R01 CA48998 to M. L. Slattery). HPFS is supported by the National Institutes of Health (P01 CA 055075, UM1 CA167552, R01 137178, R01 CA151993 and P50 CA127003), NHS by the National Institutes of Health (UM1 CA186107, R01 CA137178, P01 CA87969, R01 CA151993 and P50 CA127003) and PHS by the National Institutes of Health (R01 CA042182). MEC: National Institutes of Health (R37 CA54281, P01 CA033619, and R01 CA63464). OFCCR: National Institutes of Health, through funding allocated to the Ontario Registry for Studies of Familial Colorectal Cancer (U01 CA074783); see CCFR section above. Additional funding toward genetic analyses of OFCCR includes the Ontario Research Fund, the Canadian Institutes of Health Research, and the Ontario Institute for Cancer Research, through generous support from the Ontario Ministry of Research and Innovation. PMH: National Institutes of Health (R01 CA076366 to P.A. Newcomb). VITAL: National Institutes of Health (K05 CA154337). WHI: The WHI program is funded by the National Heart, Lung, and Blood Institute, National Institutes of Health, U.S. Department of Health and Human Services through contracts HHSN268201100046C, HHSN268201100001C, HHSN268201100002C, HHSN268201100003C, HHSN268201100004C, and HHSN271201100004C.

GECCO: The authors would like to thank all those at the GECCO Coordinating Center for helping bring together the data and people that made this project possible. The authors acknowledge Dave Duggan and team members at TGEN (Translational Genomics Research Institute), the Broad Institute, and the Génome Québec Innovation Center for genotyping DNA samples of cases and controls, and for scientific input for GECCO. ASTERISK: We are very grateful to Dr. Bruno Buecher without whom this project would not have existed. We also thank all those who agreed to participate in this study, including the patients and the healthy control persons, as well as all the physicians, technicians and students. DACHS: We thank all participants and cooperating clinicians, and Ute Handte-Daub, Utz Benscheid, Muhabbet Celik and Ursula Eilber for excellent technical assistance.

HPFS, NHS and PHS: We would like to acknowledge Patrice Soule and Hardeep Ranu of the Dana Farber Harvard Cancer Center High-Throughput Polymorphism Core who assisted in the genotyping for NHS, HPFS, and PHS under the supervision of Dr. Immaculata Devivo and Dr. David Hunter, Qin (Carolyn) Guo and Lixue Zhu who assisted in programming for NHS and HPFS, and Haiyan Zhang who assisted in programming for the PHS. We would like to thank the participants and staff of the Nurses' Health Study and the Health Professionals Follow-Up Study, for their valuable contributions as well as the following state cancer registries for their help: AL, AZ, AR, CA, CO, CT, DE, FL, GA, ID, IL, IN, IA, KY, LA, ME, MD, MA, MI, NE, NH, NJ, NY, NC, ND, OH, OK, OR, PA, RI, SC, TN, TX, VA, WA, WY. The authors assume full responsibility for analyses and interpretation of these data. PMH: The authors would like to thank the study participants and staff of the Hormones and Colon Cancer study. WHI: The authors thank the WHI investigators and staff for their dedication, and the study participants for making the program possible. A full listing of WHI investigators can be found at: [http://www.whi.org/researchers/Documents%20%20Write%20a%20Paper/WHI%20Investigator%20Short%20List.pdf](http://www.whi.org/researchers/Documents  Write a Paper/WHI Investigator Short List.pdf)

**PanScan I, II and III authors:** Brian M. Wolpin^1, 2^, Cosmeri Rizzato^3^, Peter Kraft^4, 5^, Charles Kooperberg^6^, Gloria M. Petersen^7^, Zhaoming Wang^8, 9^, Alan A. Arslan^10, 11, 12^, Laura Beane-Freeman^8^, Paige M. Bracci^13^, Julie Buring^14,15^, Federico Canzian^3^, Eric J. Duell^16^, Steven Gallinger^17^, Graham G. Giles^18, 19, 20^, Gary E. Goodman^6^, Phyllis J. Goodman^21^, Eric J. Jacobs^22^, Aruna Kamineni^23^, Alison P. Klein^24, 25^, Laurence N. Kolonel^26^, Matthew H. Kulke^1^, Donghui Li^27^, Núria Malats^28^, Sara H. Olson^29^, Harvey A. Risch^30^, Howard D. Sesso^4, 14, 15^, Kala Visvanathan^31^, Emily White^32, 33^, Wei Zheng^34, 35^, Christian C. Abnet^8^, Demetrius Albanes^8^, Gabriella Andreotti^8^, Melissa A. Austin^33^, Richard Barfield^5^, Daniela Basso^36^, Sonja I. Berndt^8^, Marie-Christine Boutron-Ruault^37, 38, 39^, Michelle Brotzman^40^, Markus W. Büchler^41^, H. Bas Bueno-de-Mesquita^42, 43, 44^, Peter Bugert^45^, Laurie Burdette^8, 9^, Daniele Campa^46^, Neil E. Caporaso^8^, Gabriele Capurso^47^, Charles Chung^8, 9^, Michelle Cotterchio^48, 49^, Eithne Costello^50^, Joanne Elena^51^, Niccola Funel^52^, J. Michael Gaziano^14, 15, 53^, Nathalia A. Giese^41^, Edward L. Giovannucci^4, 54, 55^, Michael Goggins^56, 57, 58^, Megan J. Gorman^1^, Myron Gross^59^, Christopher A. Haiman^60^, Manal Hassan^27^, Kathy J. Helzlsouer^61^, Brian E. Henderson^62^, Elizabeth A. Holly^13^, Nan Hu^8^, David J. Hunter^2, 63, 64^, Federico Innocenti^65^, Mazda Jenab^66^, Rudolf Kaaks^46^, Timothy J. Key^67^, Kay-Tee Khaw^68^, Eric A. Klein^69^, Manolis Kogevinas^70, 71, 72^, Vittorio Krogh^73^, Juozas Kupcinskas^74^, Robert C. Kurtz^75^, Andrea LaCroix^6^, Maria T. Landi^8^, Stefano Landi^76^, Loic Le Marchand^77^, Andrea Mambrini^78^, Satu Mannisto^79^, **Roger L. Milne**^18, 19^, Yusuke Nakamura^80^, Ann L. Oberg^81^, Kouros Owzar^82^, Alpa V. Patel^22^, Petra H. M. Peeters^83, 84^, Ulrike Peters^85^, Raffaele Pezzilli^86^, Ada Piepoli^87^, Miquel Porta^71, 88, 89^, Francisco X. Real^90, 91^, Elio Riboli^44^, Nathaniel Rothman^8^, Aldo Scarpa^92^, Xiao-Ou Shu^34, 35^, Debra T. Silverman^8^, Pavel Soucek^93^, Malin Sund^94^, Renata Talar-Wojnarowska^95^, Philip R. Taylor^8^, George E. Theodoropoulos^96^, Mark Thornquist^6^, Anne Tjønneland^97^, Geoffrey S. Tobias^8^, Dimitrios Trichopoulos^4, 98, 99^, Pavel Vodicka^100^, Jean Wactawski-Wende^101^, Nicolas Wentzensen^8^, Chen Wu^4^, Herbert Yu^77^, Kai Yu^8^, Anne Zeleniuch-Jacquotte^11, 12^, Robert Hoover^8^, Patricia Hartge^8^, Charles Fuchs^1, 54^, Stephen J. Chanock^8, 9^, Rachael S. Stolzenberg-Solomon^8^, Laufey T. Amundadottir^8^

^1^ Department of Medical Oncology, Dana-Farber Cancer Institute, Boston, Massachusetts, USA

^2^ Department of Medicine, Brigham and Women’s Hospital and Harvard Medical School, Boston, Massachusetts, USA

^3^ Genomic Epidemiology Group, German Cancer Research Center (DKFZ), Heidelberg, Germany

^4^ Department of Epidemiology, Harvard School of Public Health, Boston, Massachusetts, USA

^5^ Department of Biostatistics, Harvard School of Public Health, Boston, Massachusetts, USA

^6^ Division of Public Health Sciences, Fred Hutchinson Cancer Research Center, Seattle, Washington, USA

^7^ Division of Epidemiology, Department of Health Sciences Research, Mayo Clinic, Rochester, Minnesota, USA

^8^ Division of Cancer Epidemiology and Genetics, National Cancer Institute, National Institutes of Health, Bethesda, Maryland, USA

^9^ Cancer Genomics Research Laboratory, National Cancer Institute, Division of Cancer Epidemiology and Genetics, Leidos Biomedical Research, Inc., Frederick National Laboratory for Cancer Research, Frederick, Maryland, USA

^10^ Department of Obstetrics and Gynecology, New York University School of Medicine, New York, New York, USA

^11^ Department of Environmental Medicine, New York University School of Medicine, New York, New York, USA

^12^ New York University Cancer Institute, New York, New York, USA

^13^ Department of Epidemiology and Biostatistics, University of California San Francisco, San Francisco, California, USA

^14^ Division of Preventive Medicine, Department of Medicine, Brigham and Women’s Hospital and Harvard Medical School, Boston, Massachusetts, USA

^15^ Division of Aging, Department of Medicine, Brigham and Women’s Hospital and Harvard Medical School, Boston, Massachusetts, USA

^16^ Unit of Nutrition, Environment and Cancer, Cancer Epidemiology Research Program, Bellvitge Biomedical Research Institute (IDIBELL), Catalan Institute of Oncology (ICO), Barcelona, Spain

^17^ Samuel Lunenfeld Research Institute, Mount Sinai Hospital, Toronto, Ontario, Canada

^18^ Cancer Epidemiology Centre, Cancer Council Victoria, Melbourne, Victoria, Australia

^19^ Centre for Epidemiology and Biostatistics, Melbourne School of Population and Global Health, The University of Melbourne, Victoria, Australia

^20^ Department of Epidemiology and Preventive Medicine, Monash University, Melbourne, Victoria, Australia

^21^ Southwest Oncology Group Statistical Center, Fred Hutchinson Cancer Research Center, Seattle, Washington, USA

^22^ Epidemiology Research Program, American Cancer Society, Atlanta, Georgia, USA

^23^ Group Health Research Institute, Seattle, Washington, USA

^24^ Department of Oncology, the Johns Hopkins University School of Medicine, Baltimore, Maryland, USA

^25^ Department of Epidemiology, the Bloomberg School of Public Health, Baltimore, Maryland, USA

^26^ The Cancer Research Center of Hawaii (retired), Honolulu, Hawaii, USA

^27^ Department of Gastrointestinal Medical Oncology, University of Texas M.D. Anderson Cancer Center, Houston, Texas, USA

^28^ Genetic and Molecular Epidemiology Group, CNIO-Spanish National Cancer Research Centre, Madrid, Spain

^29^ Department of Epidemiology and Biostatistics, Memorial Sloan-Kettering Cancer Center, New York, New York, USA

^30^ Department of Chronic Disease Epidemiology, Yale School of Public Health, New Haven, Connecticut, USA

^31^ Johns Hopkins Bloomberg School of Public Health, Baltimore, Maryland, USA

^32^ Fred Hutchinson Cancer Research Center, Seattle, Washington, USA

^33^ Department of Epidemiology, University of Washington, Seattle, Washington, USA

^34^ Department of Medicine, Vanderbilt University Medical Center, Nashville, Tennessee, USA

^35^ Vanderbilt-Ingram Cancer Center, Vanderbilt University Medical Center, Nashville, Tennessee, USA

^36^ Department of Laboratory Medicine, University Hospital of Padova, Padua, Italy

^37^ Inserm, Centre for Research in Epidemiology and Population Health (CESP), U1018, Nutrition, Hormones and Women’s Health Team, F-94805, Villejuif, France

^38^ University Paris Sud, UMRS 1018, F-94805, Villejuif, France

^39^ IGR, F-94805, Villejuif, France

**^40^ Westat, Rockville, Maryland, USA**

^41^ Department of General Surgery, University Hospital Heidelberg, Heidelberg, Germany

^42^ National Institute for Public Health and the Environment (RIVM), Bilthoven, The Netherlands

^43^ Department of Gastroenterology and Hepatology, University Medical Centre Utrecht, Utrecht, The Netherlands

^44^ Department of Epidemiology and Biostatistics, School of Public Health, Imperial College London, London, United Kingdom

^45^ Institute of Transfusion Medicine and Immunology, Heidelberg University, Medical Faculty Mannheim, German Red Cross Blood Service Baden-Württemberg-Hessen, Mannheim, Germany

^46^ Division of Cancer Epidemiology, German Cancer Research Center (DKFZ), Heidelberg, Germany

^47^ Digestive and Liver Disease Unit, ‘Sapienza’ University of Rome, Rome, Italy

^48^ Cancer Care Ontario, University of Toronto, Toronto, Ontario, Canada

^49^ Dalla Lana School of Public Health, University of Toronto, Toronto, Ontario, Canada

^50^ National Institute for Health Research Liverpool Pancreas Biomedical Research Unit, University of Liverpool, Liverpool, United Kingdom

^51^ Division of Cancer Control and Population Sciences, National Cancer Institute, National Institutes of Health, Bethesda, Maryland, USA

^52^ Department of Surgery, Unit of Experimental Surgical Pathology, University Hospital of Pisa, Pisa, Italy

^53^ Massachusetts Veteran’s Epidemiology, Research, and Information Center, Geriatric Research Education and Clinical Center, Veterans Affairs Boston Healthcare System, Boston, Massachusetts, USA

^54^ Channing Division of Network Medicine, Department of Medicine, Brigham and Women’s Hospital, and Harvard Medical School, Boston, Massachusetts, USA

^55^ Department of Nutrition, Harvard School of Public Health, Boston, Massachusetts, USA

^56^ Department of Pathology, Sidney Kimmel Cancer Center and Johns Hopkins University, Baltimore, Maryland, USA

^57^ Department of Medicine, Sidney Kimmel Cancer Center and Johns Hopkins University, Baltimore, Maryland, USA

^58^ Department of Oncology, Sidney Kimmel Cancer Center and Johns Hopkins University, Baltimore, Maryland, USA

^59^ Laboratory of Medicine and Pathology, University of Minnesota, Minneapolis, Minnesota, USA

###### ^60^ Preventive Medicine, [University of Southern California, Los Angeles, California, USA](http://www.zoominfo.com/c/University-of-Southern-California/55968079)

^61^ Prevention and Research Center, Mercy Medical Center, Baltimore, Maryland, USA

^62^ Cancer Prevention, University of Southern California, Los Angeles, California, USA

^63^ Harvard School of Public Health, Boston, Massachusetts, USA

^64^ Harvard Medical School, Boston, Massachusetts, USA

^65^ The University of North Carolina Eshelman School of Pharmacy, Center for Pharmacogenomics and Individualized Therapy, Lineberger Comprehensive Cancer Center, School of Medicine, Chapel Hill, North Carolina, USA

^66^ International Agency for Research on Cancer, Lyon, France

^67^ Cancer Epidemiology Unit, University of Oxford, Oxford, United Kingdom

**^68^ School of Clinical Medicine**, University of Cambridge, United Kingdom

^69^ Glickman Urological and Kidney Institute, Cleveland Clinic, Cleveland, OH, USA

^70^ Centre de Recerca en Epidemiologia Ambiental (CREAL), CIBER Epidemiología y Salud Pública (CIBERESP), Spain

^71^ Hospital del Mar Institute of Medical Research (IMIM), Barcelona, Spain

^72^ National School of Public Health, Athens, Greece

^73^ Epidemiology and Prevention Unit, Fondazione IRCCS Istituto Nazionale dei Tumori, Milan, Italy

^74^ Department of Gastroenterology, Lithuanian University of Health Sciences, Kaunas, Lithuania

^75^ Department of Medicine, Memorial Sloan-Kettering Cancer Center, New York, New York, USA

^76^ Department of Biology, University of Pisa, Pisa, Italy

^77^ Cancer Epidemiology Program, University of Hawaii Cancer Center, Honolulu, HI, USA

^78^ Oncology Department, ASL1 Massa Carrara, Massa Carrara, Italy

^79^ National Institute for Health and Welfare, Department of Chronic Disease Prevention, Helsinki, Finland

^80^ Human Genome Center, Institute of Medical Science, The University of Tokyo, Tokyo, Japan

^81^ Alliance Statistics and Data Center, Division of Biomedical Statistics and Informatics, Department of Health Sciences Research, Mayo Clinic, Rochester, Minnesota, USA

^82^ Alliance Statistics and Data Center, Department of Biostatistics and Bioinformatics, Duke Cancer Institute, Duke University Medical Center, Durham, North Carolina, USA

^83^ Julius Center for Health Sciences and Primary Care, University Medical Center Utrecht, Utrecht, The Netherlands

^84^ Department of Epidemiology and Biostatistics, School of Public Health, Imperial College London, London, United Kingdom

^85^ Epidemiology, Fred Hutchinson Cancer Research Center, Seattle, Washington, USA

^86^ Pancreas Unit, Department of Digestive Diseases and Internal Medicine, Sant’Orsola-Malpighi Hospital, Bologna, Italy

^87^ Department of Gastroenterology, Scientific Institute and Regional General Hospital “Casa Sollievo della Sofferenza”, Opera di Padre Pio da Pietrelcina, San Giovanni Rotondo, Italy

^88^ School of Medicine, Universitat Autònoma de Barcelona, Spain

^89^ CIBER de Epidemiología y Salud Pública (CIBERESP), Spain

^90^ Epithelial Carcinogenesis Group, CNIO-Spanish National Cancer Research Centre, Madrid, Spain

^91^ Departament de Ciències i de la Salut, Universitat Pompeu Fabra, Barcelona, Spain

^92^ ARC-NET: Centre for Applied Research on Cancer, University and Hospital Trust of Verona, Verona, Italy

^93^ Toxicogenomics Unit, Center for Toxicology and Safety, National Institute of Public Health, Prague, Czech Republic

^94^ Department of Surgical and Peroperative Sciences, Umeå University, Umeå, Sweden

^95^ Department of Digestive Tract Diseases, Medical University of Łodz, Łodz, Poland

^96^ 1st Propaideutic Surgical Department, Hippocration University Hospital, Athens, Greece

^97^ Institute of Cancer Epidemiology, Danish Cancer Society, Copenhagen, Denmark

^98^ Bureau of Epidemiologic Research, Academy of Athens, Athens, Greece

^99^ Hellenic Health Foundation, Athens, Greece

^100^ Department of Molecular Biology of Cancer, Institute of Experimental Medicine, Academy of Sciences of the Czech Republic, Prague, Czech Republic

^101^ Department of Social and Preventive Medicine, University at Buffalo, Buffalo, New York, USA

**MGS Consortium**

The Molecular Genetics of Schizophrenia Consortium includes P.V. Gejman, A.R. Sanders, J. Duan (North Shore University Health System and University of Chicago), C.R. Cloninger, D.M. Svrakic (Washington University, St. Louis), N.G. Buccola (Louisiana State University Health Sciences Center, New Orleans), D.F. Levinson, J. Shi (Stanford University, Stanford, Calif.; Dr. Shi is now at the National Cancer Institute), B.J. Mowry (Queensland Centre for Mental Health Research, Brisbane, and Queensland Brain Institute, University of Queensland, Brisbane), R. Freedman, A. Olincy (University of Colorado Denver), F. Amin (Atlanta Veterans Affairs Medical Center and Emory University, Atlanta), D.W. Black (University of Iowa Carver College of Medicine, Iowa City), J.M. Silverman (Mount Sinai School of Medicine, New York), and W.F. Byerley (University of California, San Francisco).
