# Supplementary figures and images for: Winner's Curse Correction and Variable Thresholding Improve Performance of Polygenic Risk Modeling Based on Genome-Wide Association Study Summary-Level Data
Source: PLoS Genet. 2016 Dec 30;12(12):e1006493. doi: 10.1371/journal.pgen.1006493 (PMC5201242; doi:10.1371/journal.pgen.1006493)

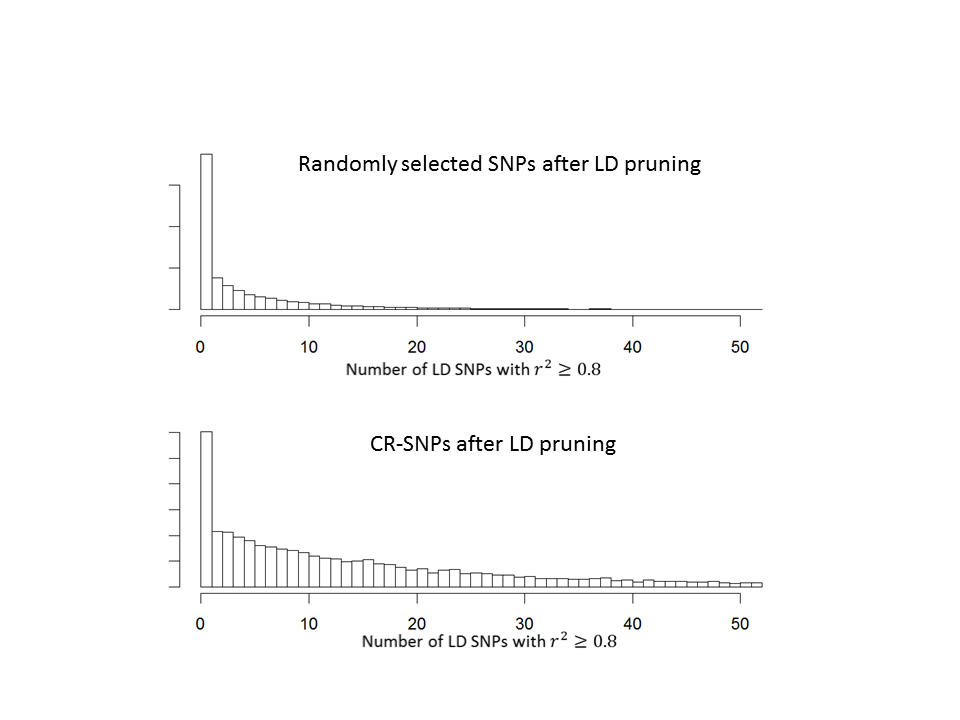

Supplement: S1 Fig — (TIF) [file pgen.1006493.s014.tif]

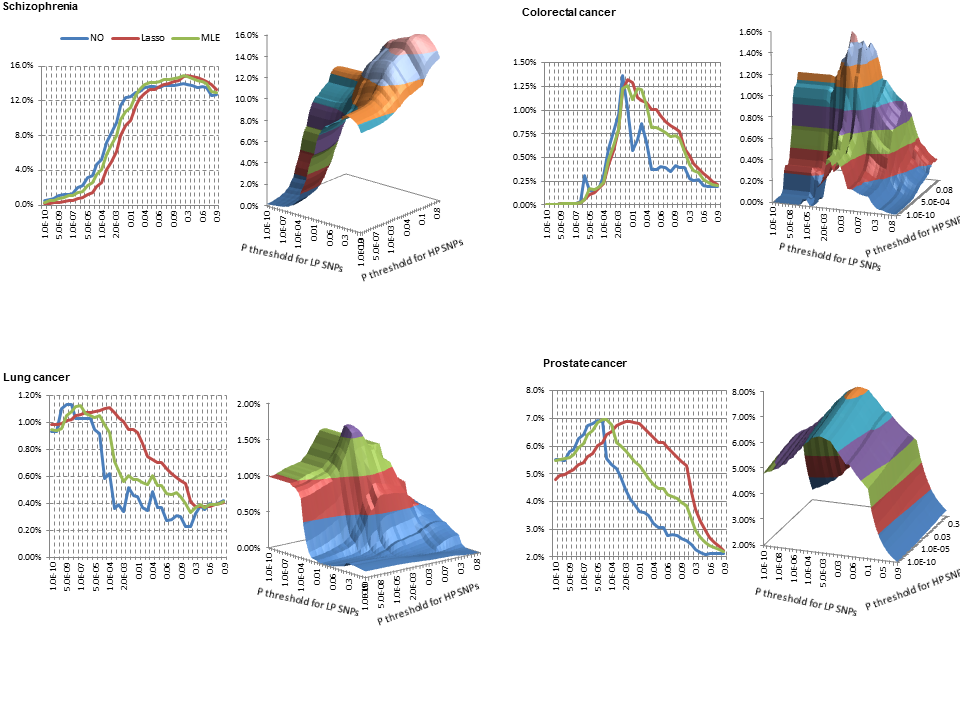

Supplement: S2 Fig — (TIF) [file pgen.1006493.s015.tif]
